# Models of Variability in Probabilistic Causal Judgments

**DOI:** 10.1007/s42113-024-00223-7

**Published:** 2024-10-08

**Authors:** Ivar Kolvoort, Zachary J. Davis, Bob Rehder, Leendert van Maanen

**Affiliations:** 1 Amsterdam University Medical Centre, University of Amsterdam, Amsterdam, The Netherlands; 2 Department of Psychology, University of Amsterdam, Amsterdam, The Netherlands; 3 Department of Psychology, New York University, New York, USA; 4 Department of Experimental Psychology, Utrecht University, Utrecht, The Netherlands

**Keywords:** Causal reasoning, Causal judgment, Cognitive modeling, Response distributions, Sampling, Repeated-measures

## Abstract

Most theories of causal reasoning aim to explain the central tendency of causal judgments. However, experimental studies show that causal judgments are quite variable. In this article, we report the results of an experiment using a novel repeated measures design that demonstrate the existence of meaningful (i.e., not noise-related) within-participant variability. Next, we introduce and assess multiple computational cognitive models that serve as potential accounts of the sources of variability and fit those models to the new empirical data. We find that the *Bayesian Mutation Sampler* has the best fit to the data and is able to account for a number of unusual features of the response distributions (e.g., bi-modality), supporting the view that the stochastic sampling mechanism it posits reflects the cognitive processes via which people draw causal inferences. Additionally, our findings suggest that incorporating ‘non-reasoning’ processes, such as rounding and guessing, can improve the ability of models of causal reasoning to account for the observed response distributions. Overall, the study highlights the potential of computational modeling of full response distributions to shed light on the underlying mechanisms of human causal reasoning and identifies promising directions for future research.

**Supplementary Information:**

The online version contains supplementary material available at 10.1007/s42113-024-00223-7.

## Introduction

One important way in which we understand the world is through the lens of causation. Our knowledge about causality in our environment has been found to affect a myriad of decisions and judgments (see Danks, 2014; Sloman, 2005; Sloman & Lagnado, 2015; M R Waldmann, 2017). Over the last decades a renewed interest in causal cognition has led to a wealth of studies investigating different facets of causal cognition. One of the main tools used to understand human causal cognition is a theoretical framework known as causal Bayesian networks^1^ (CBNs; Pearl, 2009; Spirtes et al., 2000). As a normative theory CBNs have provided a decent approximation of human behavior and provided researchers with a benchmark with which to compare human behavior. But although CBNs provide a computational account that can formally describe people’s causal judgments, by themselves they do not provide insight into *how* those judgments are made. However, it is of great interest to cognitive scientists and psychologists to understand the cognitive processes that lead to these sophisticated judgments.

Rather than understanding the ‘how’ question, most past research has focused on describing *what* people are doing. This research has led to identifying many behavioral patterns in people’s causal judgments, which are often described as systematic deviations of mean judgments from the CBN predictions (Rehder, 2014; Rottman & Hastie, 2014, 2016). The many explanations for these deviations that have been offered (e.g.: Mistry et al., 2018; Rehder, 2014; Rottman & Hastie, 2016; Tešić et al., 2020; Trueblood et al., 2017), are mostly descriptive and, since they all target the same phenomena, hard to distinguish empirically. One key reason for this difficulty is that by and large the field has focused on the central tendency of responses, that is, the mean. While focusing on mean responses is a principled approach and can be effective, it discards much of the information in participants’ responses. With a target explanandum as rich as human causal cognition this has led to considerable difficulty in assessing the relative success of different candidate theories (Rehder, 2014, 2018; Rottman & Hastie, 2014, 2016).

One way forward is to focus efforts on analyzing not just the mean of causal judgments but also their distributions (Kolvoort et al., 2023; O’Neill et al., 2022). Indeed, other fields have made progress by showing that variability in behavior can be informative of underlying cognitive processes. For example, in the field of judgment and decision-making the widely used evidence accumulation models (e.g. Ratcliff, 1978; Ratcliff et al., 2016), which account for the joint distribution of responses and response times, have allowed for important theoretical developments, such as an explanation of the speed-accuracy trade-off in decision-making (e.g. Bogacz et al., 2010; Katsimpokis et al., 2020; Van Maanen et al., 2011), or an understanding of specific individual differences in decision-making behavior (Ratcliff et al., 2006; van Ravenzwaaij et al., 2011). It has been known for a while that there is a substantial amount of variability in causal judgments with multiple authors commenting on this (Davis & Rehder, 2020; Rehder, 2014; Rottman & Hastie, 2016). Hence, we believe the time has come for the field of causal reasoning to exploit the variability in causal judgments and engage in the modeling of full response distributions (Kolvoort et al., 2023).

But modeling response distributions comes with its own challenges. Because past studies reporting variability have usually presented a single type of causal judgment once, it is unclear whether that variability arose from *between* versus *within*-subject sources. Factors that contribute to between-subjects variability (e.g., motivation, varying beliefs about the underlying causal structure, etc.) might be very different from those that might lead the same individual to respond differently to the same causal query when asked a second time. Thus, identifying the underlying cognitive mechanisms that generate variability requires obtaining a good estimate of that variability at the individual level. But this in turn introduces a difficult problem in experimental design, which is that repeatedly asking a participant the same judgment is likely to yield measures that are not independent. Other research areas that commonly elicit repeated measurements often have stimuli, such as random-dot motion arrays, that can be presented repeatedly without participants being aware of the repetition. In contrast, stimuli in the typical causal reasoning study are composed of discrete symbols (such as states of binary causal variables) that are susceptible to be memorized and later recognized. For example, if a participant recognizes that a causal query has been presented previously, they may tend to respond with the same answer in order to minimize cognitive effort, to be consistent, and so forth, which would lead to a much lower estimate of within-subject variability as compared to if they recompute the answer on every trial. Due to its more deliberative and conscious nature, studies of higher order cognition may be particularly susceptible to non-independent repeated measures. Indeed, storing previous judgments for future use has been proposed to be an important source of computational savings (Dasgupta & Gershman, 2021).

This article has the following structure. We first report the results of an experiment using a novel repeated measures design that, for the first time, characterizes both between *and* within-subjects variability in causal judgments. With these data in hand, we then introduce several computational models as potential accounts of those sources of variability and quantitatively assess their goodness-of-fit to the new data set. We also perform model simulations to assess each model’s ability to predict qualitative patterns (i.e. behavioral effects) of interest (Palminteri et al., 2017).

## Experiment

To elicit independent repeated judgments, we identified three ways to present participants with causal queries that are distinct from the point of view of the participant (and thus are less susceptible to memorization) but where the underlying CBN yields the same answer. First, we tested a symmetrical causal structure so that the same logical query could be asked of different variables. Second, we instantiate that structure in different content domains (different variables, verbal descriptions of causal mechanisms, etc.) that nonetheless had the same underlying parameterization (and so should yield the same answer). Finally, for a given query, we asked subject to separately judge both the presence and absence of a variable in the causal structure (yielding two judgments for the query by subtracting the latter judgment from 1). Together, these experimental techniques resulted in obtaining from each participant 20 judgments for logically equivalent causal judgments, providing the new data needed to compare different theoretical accounts of within-subject variability.

### Materials

**Fig. 1:**
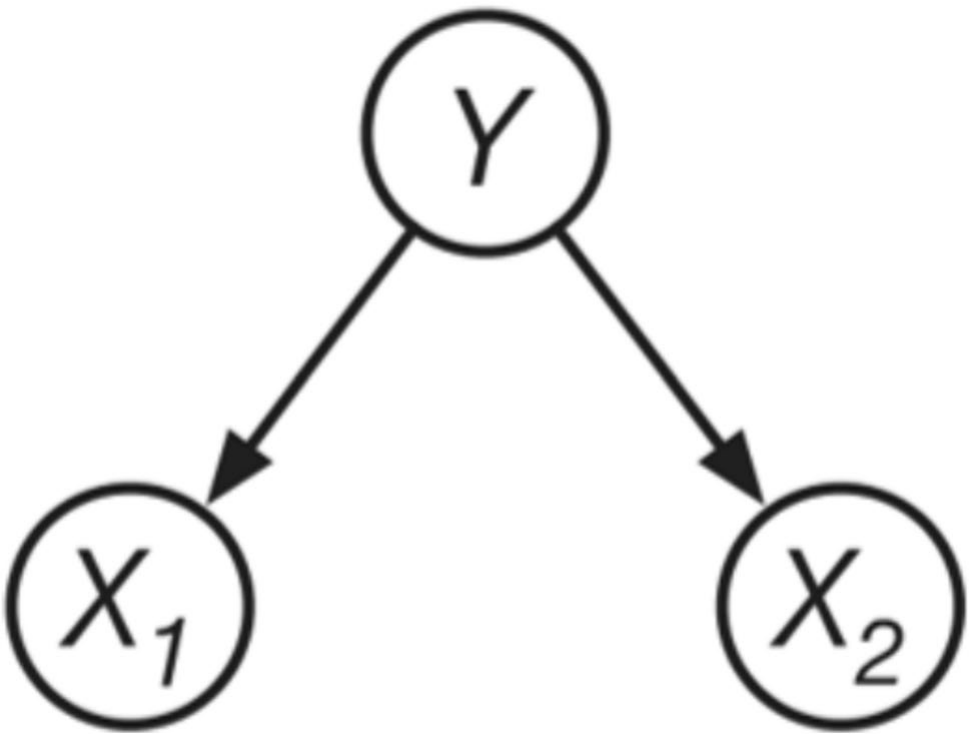
Three-variable common cause network. Arrows denote causal relationships and circles denote binary causal variables

Probabilistic causal judgments were tested in five domains: biology, sociology, astronomy, meteorology, and sociology. Participants were first told that the causal network in the domain they were about to study had three binary variables. Next, they were presented with a verbal description of two causal relationships that formed a common cause network (Fig. 1) where two variables were effects (henceforth X_1_ and X_2_) and one was the cause (Y). For each causal relationship, a description was provided that included a discussion of the generative mechanism responsible for that relationship (see https://osf.io/dpwg6/ for examples of the materials used). All the causal variables and relationships were counterbalanced over participants. The domains and descriptions were all based on standard materials that have been used and validated by multiple other studies in the field (Rehder, 2014, 2018; Rehder & Hastie, 2001; Rehder & Waldmann, 2017).

### Procedure

Subjects first studied several screens of information about the overall task that established the domains being studied and the types of inferences that would be presented during the study using a trial domain about mechanics to illustrate all elements of the inference test. Then, for each domain, initial screens presented a cover story and a description of the domain’s three variables and subsequent screens presented the two causal links and a diagram of those links. A common cause network was used in every domain, and participants were informed that each variable’s base rate was 50% and that each cause produced its effect “75% of the time”.

When ready, participants were asked three multiple-choice questions to assess their understanding of the causal relationships. This comprehension check established that they had learned which variables were causally related, the direction of those relationships, and that the relationships were probabilistic rather than deterministic. Participants were given three attempts to answer all questions correctly. Once they answered all questions correctly or after the third attempt, participants could continue with the experiment.

**Fig. 2:**
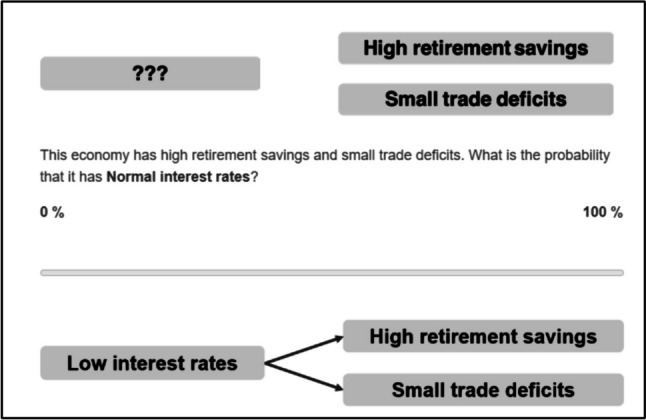
Screenshot of a trial in the experiment. This screenshot is of the $P\left(Y=1|{X}_{i}=1,{X}_{j}=1\right)$ inference. Participants respond by clicking on the horizontal scale ranging from 0 to 100%. The bottom of the screen displayed the causal network on which participants were instructed to reduce memory load (see Rehder, 2018)

Subjects were then presented with the inference test. Each trial presented the values of one or two variables and asked the subject to predict the state of another. For example, a subject might be told that an economy has low interest rates and a normal trade deficit and be asked the probability of it having a high level of retirement savings. A second example trial is presented in Fig. 2. Subjects entered their response by moving a tick on a rating scale whose ends were labeled 0% and 100%. As an attention check, participants were asked a comprehension check question at the end of each block. The order of the five domains, and the 24 test questions within each domain, was randomized for each participant.

### Design and Participants

Six different inference types were tested that varied on two factors: Information and Direction (Table 1). Direction referred to the direction of reasoning required, from cause to effect (Predictive) or from effect to cause (Diagnostic). Information refers to the variable values that are provided to the participants on each trial, which could either be Consistent (two variables with the same value), Inconsistent (two variables with differing values), and Incomplete (one variable).

**Table 1:** Inference types and normative answers. Inference types varied with two factors, Direction (predictive or diagnostic) and Information (consistent, incomplete, or inconsistent) resulting in 6 inference types. Xs and Ys refer to variables, where the Xs are effects, and the Y is the cause in a three-variable common cause network (see Fig. 1). The 1s and 0s refer to the presence or absence of an effect or cause

		Direction	
		Predictive	Diagnostic
Information	Consistent	$P\left({X}_{i}=1|Y=1,{X}_{j}=1\right)$	$P\left(Y=1|{X}_{i}=1,{X}_{j}=1\right)$
		= .80	= .94
	Incomplete	$P\left({X}_{i}=1|Y=1\right)$	$P\left(Y=1|{X}_{i}=1\right)$
		= .80	= .80
	Inconsistent	$P\left({X}_{i}=1|Y=1,{X}_{j}=0\right)$	$P\left(Y=1|{X}_{i}=1,{X}_{j}=0\right)$
		= .80	= .50

Within each of the five domains, participants responded to four different versions of each of the six inference types, resulting in 24 trials per domain. Because of the symmetry inherent in the common cause network in Fig. 1, and because all variables were described as having the same base rate and all causal relations as having the same strength, for every query that asked a participant to predict X_1_ an analogous query asking them to predict X_2_ should yield the same answer (e.g., $P\left({X}_{1}=1|Y=1\right)=$ $P\left({X}_{2}=1|Y=1\right)$). In addition, each query could ask for the probability of a variable’s presence or its absence (e.g., $P\left({X}_{1}=1|Y=1\right)$ or $P\left({X}_{1}=0|Y=1\right)$). Taken together, these methods resulted in (5 domains × 4 versions =) 20 repeated measurements for each inference type.

It is noteworthy that all the predictive inferences have the same normative probability of 80%, a result that reflects the key property of CBNs known as the *Markov condition*. Applied to the common cause network of Fig. 1, this means that X_1_ or X_2_ are independent conditioned on knowledge of the state of Y, a phenomenon also referred to as “screening off.” Specifically, according to the Markov condition

$$P\left({X}_{i}=1|Y=1,{X}_{j}=1\right)=P\left({X}_{i}=1|Y=1\right)= P\left({X}_{i}=1|Y=1,{X}_{j}=0\right)$$

should obtain. In fact, numerous studies (e.g. Ali et al., 2011; Davis & Rehder, 2020; Kolvoort et al., 2024; Mayrhofer & Waldmann, 2015; Park & Sloman, 2013, 2014; Rehder, 2014, 2018; Rehder & Waldmann, 2017; Rottman & Hastie, 2014, 2016; Sloman & Lagnado, 2015; Waldmann et al., 2008) have shown that humans’ predictive judgments are influenced by the value of the other X, exhibiting the pattern

$$P\left({X}_{i}=1|Y=1,{X}_{j}=1\right)>P\left({X}_{i}=1|Y=1\right)> P\left({X}_{i}=1|Y=1,{X}_{j}=0\right)$$

This response pattern has been referred to as “Markov violations.” The current experiment will provide another opportunity to assess for the presence of Markov violations, which provide another key data point for assessing the computational models that follow.

All participants made all judgments for all five domains. 37 participants were recruited from Prolific (www.prolific.co) and received £5.70 for on average 47 min (*SD* = 20.1) of participation. 8 (22%) participants were removed from analyses for failing at least two attention checks, as had been established by the authors before the running of the study.

### Analyses

To analyze within-participant variability as a dependent variable, we will use Gini’s Mean Difference (GMD; David, 1968; Yitzhaki, 2003) as an index of variability, which is defined as the average distance between any two observations. We use GMD instead of the parametric standard deviation as responses tend not to be normally distributed. We compute GMD for each participant separately per inference type, that is, we compute it based on 20 observations. Next, we analyze the effects of Direction and Information using repeated measures ANOVAs (using *F*-tests with Satterthwaite’s approximation). In addition to *p*-values, we compute Bayes Factors using the BayesFactor package in R with default JZS priors (Morey & Rouder, 2014) using the same comparison for the *F*-tests. For post hoc comparisons, we use Tukey’s HSD to correct *p*-values for multiple comparisons.

As the different domains as well as time-on task effects such as fatigue could possibly influence the results, we conduct additional ANOVA analyses including domain and block as factor to assess their effects on within-participant variability. However, for these analyses, we have to compute GMD based on only 4 observations (as it has to be computed per participant, inference type, and domain or block) and so we also look at the effect of domains and fatigue on the mean response. For this, we conduct similar repeated measures ANOVAs. It is important to note here that we do not use these analyses of the mean responses to establish qualitative patterns as means do not provide a good characterization of multimodal response distributions (e.g., Davis & Rehder, 2020; Kolvoort et al., 2023; Rottman & Hastie, 2016) Hence, we do not build upon these analyses and recommend, again, that researchers look towards modeling full response distributions and not just analyzing means. We conduct such modeling in later sections of this paper.

### Results

**Fig. 3:**
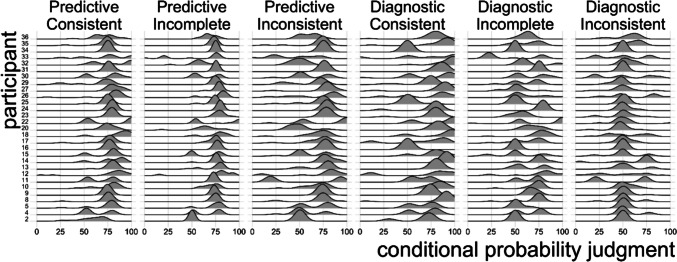
Per participant distributions of responses for each inference type. Rows correspond to participants, columns correspond to judgment types, the x-axis indicates the responses in percentage points, and the height corresponds to kernel density estimate of participant responding at this probability. Each density plot is based on 20 responses

Recall that our experimental design utilized multiple sources of redundancy to maximize the number of observations of a single causal inference. The results were collapsed over these factors to yield a total of 20 judgments per inference types per participant.^2^ Figure 3 plots the individual response distributions per inference type. This plot shows substantial between-participant variability, as we see that some participants’ responses are more spread out than others, and some participants exhibit bimodality in some or most judgments whereas others do not at all.

**Fig. 4:**
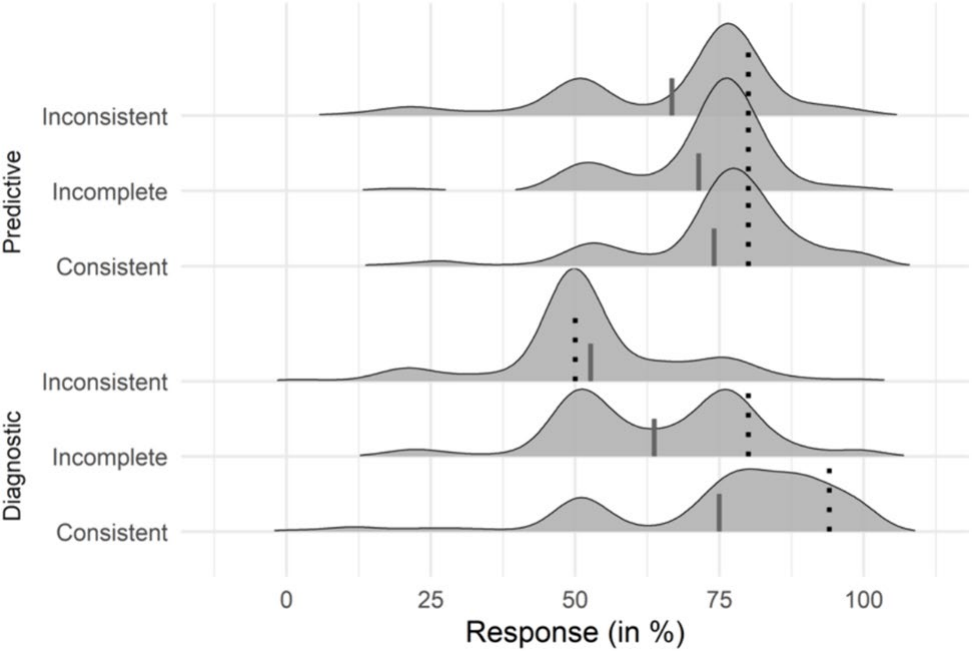
Overall response distributions per inference type. Vertical grey lines indicate mean responses. Dotted vertical black lines indicate normative response

Importantly, Fig. 3 also reveals the presence of substantial variability within each participant’s responses. Moreover, the overall spread and modality of the response distributions differs per inference type for many participants. To evaluate this effect in greater detail, Fig. 4 presents the response distributions for each inference type over all participants. One notable aspect of these data is that the distributions vary by judgment type. If the only source of variability is unrelated to the process by which causal judgments are generated (such as general response noise), we would expect similar variability across judgments.

The bimodality of the response distributions in Fig. 4 is also noteworthy. In particular, we observe a “spike” of responses at 50%, which has been reported previously (Rottman & Hastie, 2016). This peak at 50% seems to vary along the Information factor, with the largest peaks for inconsistent inferences and smallest for inferences with consistent information. As expected, the peak is largest for inconsistent diagnostic inferences for which the normative answer is 50%.

**Fig. 5:**
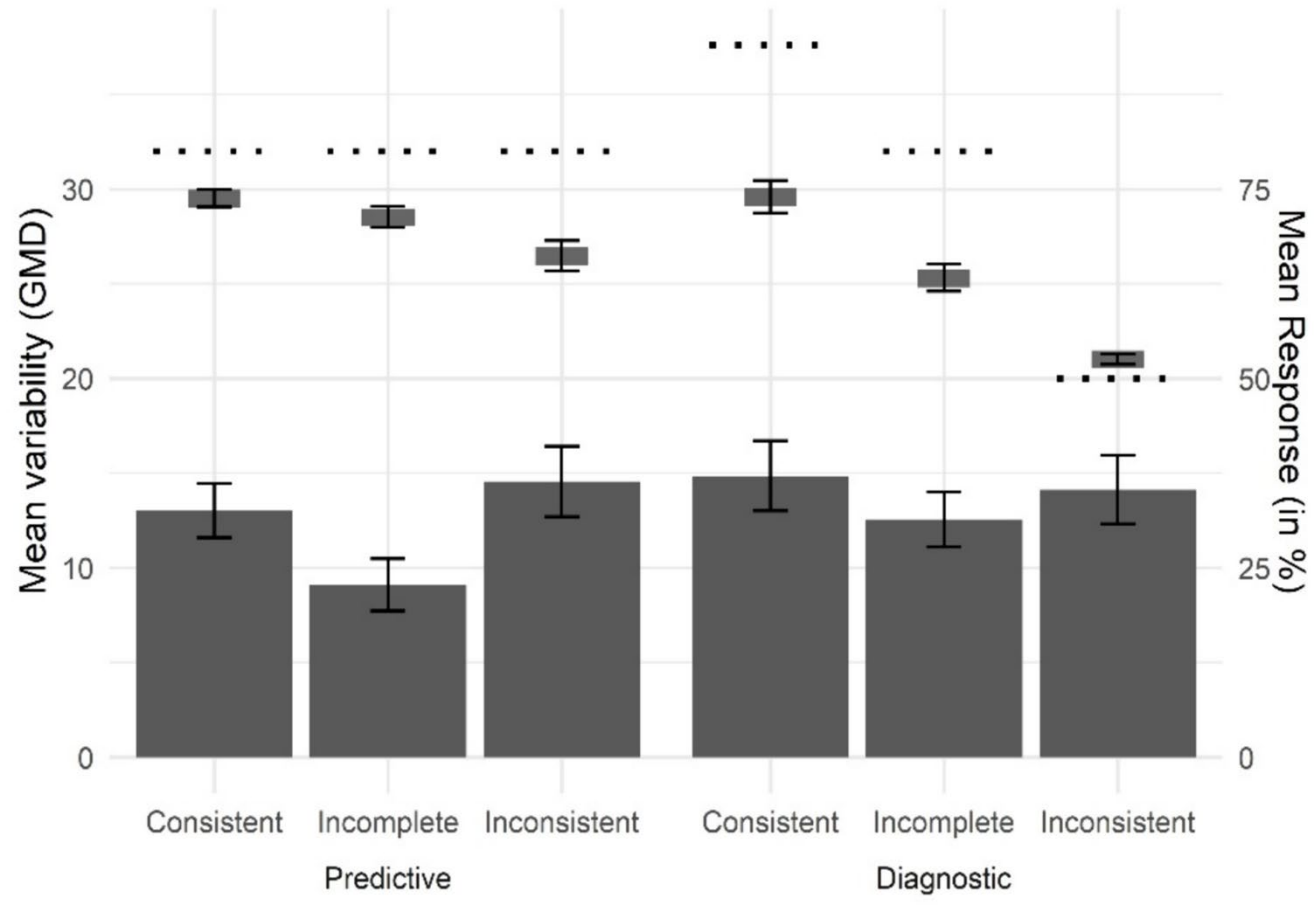
Mean and GMDs of responses per inference type. Barplot: Mean within-participant standard deviations per inference type. Floating dashes: Mean responses per inference type. Black vertical lines indicate standard error. Horizontal dotted lines indicate normative probability. GMD stands for Gini’s Mean Difference

Figure 5 shows the means of within-participant GMD and mean judgments per inference type. Note in Fig. 5 that while variability differs by inference type, it does not track with the mean, suggesting that these results are not driven by an artifact of the scoring system. We tested whether variability differs over the inference types using a repeated measures ANOVA with the GMD of responses as the dependent variable and Direction (predictive vs. diagnostic) and Information (consistent, incomplete, vs. inconsistent) as factors. The main effect of Information was significant (*F*(2,140) = 8.14, *p* < 0.001, *BF*_*10*_ = 42.6) reflecting that variability was lower for inferences with incomplete information (*Mean* = 10.8, *SE* = 1.5), than for those with complete information (*consistentMean* = 13.9, *SE* = 1.5, *inconsistentMean* = 14.3, *SE* = 1.4). We found mixed evidence of an effect of Direction (*F*(1,140) = 4.32, *p* = 0.040, *BF*_*10*_ = 0.957): Variability was marginally higher for diagnostic inferences (*Mean* = 13.8, *SE* = 1.45) than for predictive inferences (*Mean* = 12.2, *SE* = 1.45). There was no evidence for a Direction × Information interaction (*F*(2,140) = 2.08, *p* = 0.129, *BF*_*10*_ = 0.818). Note that the differences in variability over inference types suggest that variability results from the underlying cognitive process that generates causal judgments.

We also asked if variability was related to the content domain in which subjects were tested. Although the common cause structure in each of the five domains was parameterized identically, it is conceivable that subjects viewed, for example, the causal relations in one domain as more plausible than another, a factor that may have contributed to the within-participant variability in Fig. 5. To test whether the domain affected variability, we conducted a repeated measure ANOVA on the within-participant GMD with inference type and domain as factors. We found evidence against an effect of domain on variability (*F*(4,789) = 1.04, *p* = 0.385, *BF*_*10*_ < 0.01) and against an interaction of Domain with inference type (*F*(20,789) = 1.05, *p* = 0.398, *BF*_*10*_ < 0.01). We conducted a similar ANOVA to test for Domain effects on the mean response and found no such effect (Main effect: *F*(4,809) = 1.82, *p* = 0.124, *BF*_*10*_ = 0.019; interaction with inference type: *F*(4,809) = 0.711, *p* = 0.817, *BF*_*10*_ < 0.01). That content domain had negligible effects on subjects’ inferences indicates that our experimental strategy of producing repeated measures by presenting logically equivalent queries in different domains is a reasonable one.

Finally, we asked whether the observed variability was related to fatigue effects, rather than the reasoning process itself. We conducted a repeated measures ANOVA on the within-participant GMD using the order of blocks as presented as a predictor. We found a significant effect of block order (*F*(4,112) = 4.04, *p* = 0.00424, *BF*_*10*_ = 7.49). Post hoc contrasts revealed that the first block is significantly different from the latter blocks, which do not differ from each other (*Mean GMDs*: first block 19.9, second 17.1, third 16.7, fourth 16.2, fifth 16.5). That variability stayed constant after the first block suggests that it is unlikely to be due to fatigue. This result also argues against strategy changes over the blocks, which indicates that we largely succeeded in eliciting independent repeated judgments. For example, one would expect a decrease in variability over the latter blocks had subjects recognized that they were repeatedly being asked the same judgment type and so settled on a consistent response strategy. A similar analysis testing fatigue effects on mean responses reveals the exact same pattern. There was a significant effect of block order on the mean response (*F*(4,112) = 5.74, *p* < 0.001, *BF*_*10*_ = 86), which is due to the first block being different from the latter blocks, which did not differ from each other (Mean responses: first block 62.6, second 67.2, third 68.4, fourth 68.5, fifth 69.1). Moreover, a supplementary analysis treating the first block as “burn-in” indicates that the change from the first block to the latter ones does not affect our results (see Appendix 1). Lastly, it seems that possible remaining dependence in judgments, due to querying the absence versus presence of a variable, or due to switching around X_1_ and X_2_, does not affect our results (see Appendix 1).

Although the primary motive of the present study was to assess variability, it is also important to note that the present data replicated two hallmark features of human causal reasoning data. The first one is Markov violations. As mentioned, Markov violations refer to the non-adherence to the Markov property of CBNs, which stipulates that, for the common cause network tested in the current experiment, all three predictive inferences in Table 1 should be assigned the same probability (normatively, 80%). It is clear that from the top half of Fig. 5, the current study replicated the pattern of Markov violations found in many previous studies, namely that $P\left({X}_{i}=1|Y=1,{X}_{j}=1\right)>P\left({X}_{i}=1|Y=1\right)>P\left({X}_{i}=1|Y=1,{X}_{j}=0\right)$. In fact, within the predictive inferences, we found a significant main effect of Information (*F*(2,1575) = 29.6, *p* < 0.001, *BF*_*10*_ > 100), confirming the presence of Markov violations.

A second hallmark feature of causal probabilistic reasoning is conservatism (Kolvoort et al., 2024; Phillips & Edwards, 1966; Rottman & Hastie, 2014, 2016), which refers to the tendency of people to avoid extreme responses, shifting their response towards the middle of a response scale (50% in this study). Examination of Fig. 4 indicates that participants’ mean responses fell between 50% and the normative response, reflecting conservatism. The figure indicates that responses are not only conservative on average but rather that the bulk of people’s individual responses are conservative (Kolvoort et al., 2023, 2024; Rottman & Hastie, 2016).

### Discussion

This experiment yielded several theoretically important results, ones that potentially distinguish alternative computational models. First, we confirmed the presence of substantial within-participant variability in causal reasoning. Although variability has been reported previously, the current repeated measure design allows us to conclude that variability is a property of the responses of a single individual responding to multiple instances of the same causal inference. To our knowledge, this is the first assessment of the magnitude of within-participant variability in causal judgments.

We also found that the magnitude of within-participant variability varied with the type of causal inference: Variability was lower for inferences with incomplete versus complete information and higher for diagnostic versus predictive inferences. The presence of such systematic differences in variability supports the premise that variability in causal judgments is informative of the underlying cognitive processes.

We established that participants’ response distributions exhibited some distinctive qualitative patterns. These distributions were typically multimodal, often with one mode at 50%. Although this pattern has been reported previously (Kolvoort et al., 2024; Rehder, 2018; Rottman & Hastie, 2016), a new finding is that the magnitude of the mode at 50% varied with inference type. Relatively more judgments were at (or close to) 50% when the information provided was less consistent (i.e., the mode was largest for inconsistent trials and smallest for consistent trials). And, the tendency to respond at 50% seemed larger for diagnostic inferences than for predictive ones.

We summarize the empirical findings from this experiment in Table 2. For each finding, the table includes citations to other empirical studies in which the phenomena were also observed. These data points are those that should be explained by the family of computational models we now consider.

**Table 2:** Overview of qualitative patterns in response distributions of causal judgments

Qualitative pattern	Explanation	Other studies
1a. Mean conservatism	Mean response tend to be between normative probability and 50%	(Kolvoort et al., 2024; Phillips & Edwards, 1966; Rottman & Hastie, 2014, 2016)
1b. ‘moderate’ conservatism	Bulk of responses lie between normative probability and 50%	(Kolvoort et al., 2023; Rottman & Hastie, 2016)
1c. Extreme responses are rare	Participants tend to avoid the extremes of the response scale, in probabilistic causal judgment tasks this is near 0% and 100%	(Davis & Rehder, 2020; Kolvoort et al., 2023; Rottman & Hastie, 2016)
2. Markov violations	Non-adherence to Markov property, which refers to the (conditional) independence of causal variables. In the case of a common cause network, this is the independence of X_i_ and X_j_ once the state of Y is known. This phenomenon is also referred to as ‘failures to screen off’	(Davis & Rehder, 2020; Kolvoort et al., 2024; Mayrhofer & Waldmann, 2015; Park & Sloman, 2013, 2014; Rehder, 2014, 2018; Rehder & Waldmann, 2017; Rottman & Hastie, 2014, 2016; Sloman & Lagnado, 2015; M R Waldmann et al., 2008)
3a. Within-participant variability is lower for incomplete information	Responses to queries with incomplete information are less variable	
3b. Within-participant variability is higher for diagnostic inferences	Judgments are more variable when participants are asked to reason from effect to cause (Diagnostic) as compared to when they reason from cause to effect (Predictive)	
4. Multi-modal response distributions	Response distributions often have more than one mode, even on the participant level	(Davis & Rehder, 2020; Kolvoort et al., 2023; Rottman & Hastie, 2016)
5a. Spikes at 50%	Response distributions often have a mode or ‘spike’ of responses at 50%	(Davis & Rehder, 2020; Kolvoort et al., 2024; Rehder, 2018; Rottman & Hastie, 2016)
5b. Spikes at 50% increase with inconsistency of information provided	Participants tend to respond at 50% less when consistent information is provided, and more when inconsistent information is provided, compared to when the information is incomplete	(Rottman & Hastie, 2016)
5c. Spikes at 50% are larger for diagnostic inferences	Participants tend to respond at 50% more so when they are asked to reason from effect to cause (Diagnostic) as compared to when they reason from cause to effect (Predictive)	

## Candidate Models

We now identify several computational models that can potentially account for the sources of variability in causal judgments along with the rest of the empirical phenomena in Table 2. Two such models—the *Bayesian Mutation Sampler* (Kolvoort et al., 2023) and the *Beta Inference Model* (Rottman & Hastie, 2016)—already exist and below we briefly describe those models and how they will be applied to the current data. We also present four additional models based on general psychological mechanisms that could possibly introduce variability: the Motor Variability Model, the Stimulus Encoding Error model, the Parameter Uncertainty Model, and the Guess model.

Note that in their presentation of the Bayesian Mutation Sampler, Kolvoort et al. (2023) showed the potential for fitting the variability of causal judgments and explaining certain qualitative response patterns (e.g., spikes at 50%). However, that study had two limitations. The first was that the data on which the models were compared did not include repeated measurements (see Kolvoort et al., 2024), limiting the conclusions about the sources of variability that could be drawn. The second was that only a very limited set of models was considered. As now demonstrated, there are multiple potential sources of variability in causal judgments and a central goal of the present article is to ask which, if any, of those sources are plausible explanations of the observed variability.

### Bayesian Mutation Sampler (BMS)

The Bayesian Mutation Sampler (BMS; Kolvoort et al., 2023) is a generalization of the Mutation Sampler (MS; Davis & Rehder, 2017, 2020; Rehder & Davis, 2021), specially developed to understand variability in causal judgments. The MS is a model of causal reasoning that assumes that individuals draw resource-constrained inferences based on a sampling process of CBNs. The model proposes that people think of concrete cases when asked to reason about a causal system. These concrete cases are states of a causal system where each variable is instantiated with a value (e.g. [X_1_ = 1, Y = 1, X_2_ = 0]) and they are retrieved from memory or generated using an internal generative model. The MS assumes people sample these cases using a Metropolis–Hastings algorithm, which is a Markov chain Monte Carlo sampling method for approximating probability distributions (Hastings, 1970; van Ravenzwaaij et al., 2018). This method converges to the true distribution when the number of samples grows large. The MS, however, assumes that people are restricted in the number of samples they can take as they are restricted in cognitive resources. This limited sampling in combination with two other assumptions is what makes the MS accurate in predicting mean responses on causal judgment tasks (Davis & Rehder, 2017, 2020; Rehder & Davis, 2021). The first assumption is that the proposal distribution for each step of sampling consists only of those states that differ from the current state by only one variable (i.e., the current state is ‘mutated’). This assumption implements the idea that when people think of a next case, that case is likely similar to the one they are thinking of currently. The second additional assumption is that the sampling process starts out at a prototypical state, which is a state in which all causal variables are either present or absent (in our case either [X_1_ = 0, Y = 0, X_2_ = 0] or [X_1_ = 1, Y = 1, X_2_ = 1]), as these states readily come to mind. Because the MS posits that people only take a limited number of samples, the proposal distribution and starting point bias the sampling process such that approximated distribution assigns more (less) probability weight to states with consistent (inconsistent) variable values than the normative distribution (see for more details Davis & Rehder, 2020). The size of this bias depends on the number of samples—the chain length—which is a free parameter of the model.

After generating a chain of samples, the relative frequencies of the obtained samples in the chain are used to estimate the probability query. For example, if we obtained the two identical samples [A = 1, B = 1] and [A = 1, B = 1], we would estimate $P\left(A=1 \right|B=1)$ as 100%, since in our set of samples in all cases where B = 1 we have that A = 1. However, it can be the case that our samples do not contain the states needed to compute the right relative frequency. For example, if we would want to estimate $P\left(A=1 \right|B=1)$, but all the states in our chain of samples have that B = 0. In such a case the MS defaults to responding with 50%.

While the MS has been shown to be able to predict mean responses (Davis & Rehder, 2020; Rehder & Davis, 2021), it failed to predict the observed response distributions (Kolvoort et al., 2023). The BMS, instead of computing judgments directly from frequencies in the obtained samples, combines the information gained from sampling with generic prior information to generate a judgment. The integration of prior information yields a better explanation of response distributions (Kolvoort et al., 2023). The BMS has two free parameters, the chain length and the β prior parameter that determines the shape of the symmetric Beta distribution used as a prior. We implemented only the BMS as it generalizes the MS, i.e. when the β prior parameter of the BMS is 0, the model is equivalent to the MS.

### Beta Inference Model (BIM)

Rottman and Hastie (2016) proposed a model of causal inference called the Beta Inference Model (BIM) to explain Markov violations and variability in judgments. The motivation for the BIM was that when in an experiment, participants are asked to learn about a causal system by experience (i.e. by viewing samples of data from the causal system, not reading descriptions of the causal system as in the repeated-measures experiment), then it is possible to compute judgments directly from the samples of data provided for learning. The BIM considers an inference such as $P\left({X}_{i}=1|Y=1\right)$, to be a problem of computing the proportion of times that X_j_ = 1 (“win”) versus X_j_ = 0 (“failure”) within the set of cases where Y = 1. The posterior distribution of that proportion is then given by a Beta distribution which describes participant judgments (Rottman & Hastie, 2016). In this sense, the model proposes that people infer a posterior distribution directly from the data they viewed to learn about the causal system (by regarding the “wins” and “failures” in the learning data), and then sample from this distribution when making the inference (Rottman & Hastie, 2016). As the learning data provided to participants represents underlying joint distributions truthfully, the mode of the predicted distributions of responses coincides with the CBN point predictions. However, the skewness and concentration of the predicted Beta distribution changes depending on the amount of learning data, which is directly related to the conditional statement, in our example Y = 1. Differences in skewness (due to differences in the conditional statement) can for example explain Markov violations as the mean of responses can shift away from the mode (which is fixed at the location of the normative probability).

As discussed, the BIM as originally proposed assumes that individuals learn about a causal structure by viewing data. However, the behavior it accounts for, such as Markov violations, have been found in experiments that do not use such a learning-by-experience procedure (Rehder & Waldmann, 2017). The BIM can be implemented without learning data by assuming that samples come from an internal generative model (cf. Rottman & Hastie, 2016), similar to the BMS. This generalized version of the BIM assumes that people generate a varying number of samples in particular ratios via their generative model.^3^ The number of samples is treated as a free parameter in the model.

Note that this approach implies that the number of samples that contribute to a given inference varies with its type. For example, the conditional probability $P\left({X}_{i}=1|Y=1\right)$ is computed on the basis on samples in which Y = 1 whereas $P\left({X}_{i}=1|Y=1,{X}_{j}=1\right)$ is computed from those in which Y = 1 and X_j_ = 1 are true. Because the former will therefore usually be computed from a larger number of samples than the latter (because there are usually more cases in which Y = 1 than there are cases in which X_j_ = 1 is also true), the Beta distribution reflecting its predictions should be more concentrated. In fact, the ratio of the concentration of the former and later inference should reflect the ratio between $P(Y=1)$ and $P\left(Y=1, {X}_{j}=1\right)$. Table 3 summarizes the relative concentration of each inference types for a common cause network under the parameterization participants were taught. Note that the assumption that the concentration parameter varies with inference type can be interpreted as reflecting that, for a given inference, people will generate more/fewer samples as function of how easy/hard they are to generate (which in turn is determined by whether they are of high/low probability). This process is analogous to what is proposed by the (B)MS to govern sample generation (cf. Davis & Rehder, 2020; Kolvoort et al., 2023).

**Table 3:** These scaling factors determine the relative concentrations of the predicted response distributions. These particular factors are derived from the causal network taught to participants in the current repeated-measures experiment

Concentration scaling for Beta Inference model			
Inference	Total samples in possible learning data	Probability of sampling from generative model	Concentration scaling factor
$P\left({X}_{i}=1|Y=1\right)$	$N(Y=1)$	$P\left(Y=1\right)=.5$	5
$P\left({X}_{i}=1|Y=1,{X}_{j}=0\right)$	$N(Y=1,{X}_{j}=0)$	$P\left(Y=1,{X}_{j}=0\right)=.1$	1
$P\left({X}_{i}=1|Y=1,{X}_{j}=1\right)$	$N(Y=1,{X}_{j}=1)$	$P\left(Y=1,{X}_{j}=1\right)=.4$	4
$P\left(Y=1|{X}_{j}=1\right)$	$N({X}_{j}=1)$	$P\left({X}_{j}=1\right)=.5$	5
$P\left(Y=1|{X}_{i}=1,{X}_{j}=0\right)$	$N{(X}_{i}=1,{X}_{j}=0)$	$P{(X}_{i}=1,{X}_{j}=0)=.16$	1.6
$P\left(Y=1|{X}_{i}=1,{X}_{j}=1\right)$	$N({X}_{i}=1,{X}_{j}=1)$	$P\left({X}_{i}=1,{X}_{j}=1\right)=.34$	3.4

### Motor Variability Model (MVM)

As mentioned, we implemented four new models to account for variability in causal judgments. The *Motor Variability Model* (MVM) posits that people reason normatively, but their responses vary from trial to trial due to motor noise (or general task noise). In other domains, there is ample evidence that motor noise introduces variability in judgments (e.g., Maaß et al., 2021; Müller & Sternad, 2004; Verdonck & Tuerlinckx, 2013). The idea that people reason normatively to some extent but that such reasoning interacts with ‘non-normative’ processes to result in judgments has been proposed before (see Rehder & Waldmann, 2017; Rottman & Hastie, 2016).

Like the BIM, the MVM predicts a distribution of responses in which the mode is centered at the normative response and where noise is represented with a Beta distribution. But unlike the BIM, which predicts noise that varies with inference type, the MVM predicts the same variability for all inferences. It has only one free parameter, the concentration parameter of the Beta distribution.

### Stimulus Encoding Error Model (SEE)

The *Stimulus Encoding Error* (SEE) model is based on the idea that participants can misread part of the stimulus such that they encode a value for variable that differs from the one presented. For instance, instead of correctly encoding the stimulus equivalent of $P\left({X}_{i}=1|Y=0,{X}_{j}=1\right)$, a participant could erroneously encode the stimulus as $P\left({X}_{i}=1|Y=1,{X}_{j}=0\right)$. One reason to include such a model in our analysis is that the current study established that individual-level response distributions are multimodal. Misreading variable values on some trials, but not others, is a simple mechanism which would lead to multimodal distributions.

The SEE model assumes that a participant misreads the state of a conditioning variable with probability *m*. For inferences with one conditioning variable (e.g., $P\left({X}_{i}=1|Y=1\right)$) this means that the model predicts a probability of 1-*m* correct responses, and a probability of *m* incorrect responses (i.e., responses to $P\left({X}_{i}=1|Y=0\right)$). For inferences with two conditioning variables (e.g., $P\left({X}_{i}=1|Y=1,{X}_{j}=1\right)$) we still have that *m* is the probability of independently misreading the value of each conditioning variable. This leads to the probability of misreading the values of both variables being *m*^2^. The probability of only the first variable being misread is the same as the probability of only the second being misread, namely $m-{m}^{2}$. The probability of neither being misread is therefore $1-2m+{m}^{2}$. As misreading a variable value can lead to responses very different from the normative response this mechanism can predict multiple modes (as can been seen from the number of different possible responses per row in Table 4). Using this implementation, the SEE model has only one free parameter, the probability *m* of misreading the state of a conditioning variable.

**Table 4:** The free parameter m is the probability of misreading the state of a conditional variable. Probability of only misreading the first conditional variable state is m-m^2^, misreading second is also m-m^2^, and misreading both is m^2^. The probability of misreading neither is $1-2m+{m}^{2}$

Predictions of SEE model					
Inference	Normative prob. (misreading neither)	Possible ‘erroneous’ responses			Possible # of modes when m > 0
		Misread first	Misread second	Misread both	
$P\left({X}_{i}=1|Y=1\right)$	.8	.2	-	-	2
$P\left({X}_{i}=1|Y=1,{X}_{j}=0\right)$	.8	.2	.8	.2	2
$P\left({X}_{i}=1|Y=1,{X}_{j}=1\right)$	.8	.2	.8	.2	2
$P\left(Y=1|{X}_{i}=1\right)$	.8	.2	-	-	2
$P\left(Y=1|{X}_{i}=1,{X}_{j}=0\right)$	.5	.06	.94	.5	3
$P\left(Y=1|{X}_{i}=1,{X}_{j}=1\right)$	.94	.5	.5	.06	3

### Parameter Uncertainty Model (PUM)

The *Parameter Uncertainty Model* (PUM) posits that another possible source of within-participant variability is uncertainty regarding the causal network’s parameters, i.e. the base rates^4^ and causal strength parameters. Such uncertainty could lead a reasoner to use slightly different values for these parameters every time a response is computed. Because — according to CBN — not all causal parameters are necessary to compute every inference, the amount of variability the PUM predicts thus varies per inference type. For instance, diagnostic inferences require information regarding the strength of background causes for both the cause and effect, while predictive inferences only require information regarding the background causes of the effect.

We model parameter uncertainty by first drawing the causal parameters (base rates, causal strengths) from a Beta distribution centered on the normative parameter value. Next, these noisy causal parameters are used to compute a judgment according to the normative CBN framework. While the Beta distribution has its mode at the normative probability of the parameter, the concentration of the distribution can vary as a free parameter to model different levels of uncertainty. Moreover, as the causal parameters are of two types, base rates and causal strengths, each type has its own concentration parameter to model possible reasoners that are more uncertain about one type than the other. Consequently, the PUM has two free parameters, namely the concentration parameters for the Beta distributions from which the base rates and causal strengths will be sampled.

### Mixture Modeling Using a Guess Component

One thing to note from the above is that, because they predict the same distribution of responses for each of the predictive inference types in Table 1, the SEE, MVM, PUM models do not produce Markov violations. Given that evidence for Markov violations is so widespread, we also combined these models (and indeed the BMS and BIM as well) with a *Guess Model*, which stipulates that the participant responds with 50% on some proportion of trials (Rottman & Hastie, 2016). Guessing explains Markov violations assuming the guessing rate varies with the type of inference. Of course, it can also help explain the large spike of responses at 50% observed in the current data and elsewhere (Kolvoort et al., 2023; Rottman & Hastie, 2016).

There are many possible reasons why a participant would guess, such as a lack of task compliance, a lapse in concentration, or not understanding the stimulus (e.g. in case of inconsistent trials) and indeed the fact that participants report less confidence in responses near 50% (Kolvoort et al., 2024) suggests that guessing might not be uncommon. Incorporating a guessing component into BMS, BIM, MVM, SEE, and PUM (which we will refer to as ‘base’ models) makes their resultant predictions a mixture of a guessing and reasoning.

As previously summarized, the magnitude of spikes at 50% varied with the type of inference. They were larger for inconsistent inferences, which might be interpreted as eliciting more guessing because of their conflicting cues. They were also larger for diagnostic inferences, which other researchers have noted that participants find more difficult than predictive ones (Fernbach & Darlow, 2010; Fernbach et al., 2011). Because guessing might thus vary with inference type, we implemented five different Guess models: one with 0 parameters (no guessing), 1 (fixed guessing rate for all inference types), 2 (one guessing rate for predictive inferences, another for diagnostic inferences), 3 (one guessing rate each for consistent, incomplete, and inconsistent inferences), and 6 (a different rate for each inference type).

## Fitting and Analysis Methodology

### Fitting Procedure

To fit the theoretical models described above to the data we use a simulation-based approach combined with an exhaustive grid search. This is a form of ‘pre-paid’ estimation (Mestdagh et al., 2019), which falls under the umbrella of amortized inference methods (Radev et al., 2020). We use such a grid search as it removes possible bias in the optimization procedure. Such a bias has been found to be severe for fitting the Mutation Sampler and Bayesian Mutation Sampler models with a step-wise optimization procedure (Kolvoort et al., 2023). We will fit the models to each participant separately. As we keep parameters fixed for the base models over the different inference types in the experiment, this means that we will fit each set of parameters to (6 inference types × 20 repeated measurements =) 120 responses.

In the first step of the fitting procedure, we simulate responses using the models and save these simulated responses in a grid. We choose a range of realistic parameters (see below) for these simulations so that the grid covers plausible response distributions under the different models. We will simulate 100,000 responses for every combination of parameters for each model. Next, we use Probability Density Approximation (PDA; Holmes, 2015; Turner & Sederberg, 2014) to construct a ‘synthetic’ likelihood for each cell in the grid by way of kernel density estimation. This provides a likelihood of observing the data under each model and set of parameters, for each cell of the grid. The best fitting model and parameters for each participant are then given by the cell with the highest likelihood. One important parameter setting for the PDA method is the kernel bandwidth (see Lin et al., 2019). We picked our bandwidth based on the dataset, so that the predicted distributions will match the granularity of responses. To do this, we applied the Sheather and Jones method for non-parametric automatic bandwidth selection (Jones et al., 1996; Sheather & Jones, 1991) to each participant’s data and averaged those. This average was 2.13 (on percentage point scale), which we used as the standard deviation of a gaussian kernel for the kernel density estimation (cf. Lin et al., 2019). In the next step, we compute BIC values and weights (Schwarz, 1978; Wagenmakers & Farrell, 2004) to compare the fit of the models for each participant.

### Determining Grid Dimensions

We aimed to have 20 values^5^ for each free parameter of the main models and 7 values for the Guess model component (see below), to cover the range of plausible values. This number of parameter values is restricted due to computational resources related to the combination of the main models with the guess proportions. For the BMS, previous work has indicated that it is more important to have a non-biased optimization procedure than to have a very precise parameter estimate as small differences in parameter estimates only lead to small differences in the predictions (see Appendix 1 in Kolvoort et al., 2023).

The BIM, MVM, and PUM make use of Beta distributions in their implementation. For these Beta distributions, we do not use the standard parametrization in terms of shape parameters, but rather use an alternative parametrization in terms of the concentration and mode to define the distributions (Johnson et al., 1995). For these models, the concentration is a free parameter for which we will pick grid values. The other parameter, the location of the mode, is fixed by the model specification (at the normative response for the BIM and MVM, and at the normative causal strength and base rate values for the PUM).

The BMS and PUM have two free parameters and the BIM, MVM and SEE have one. Additionally, the Guess Model has a maximum of 6 parameters (one guess proportion for each of 6 inference types when it is allowed to vary for both Direction and Information factors). Following this, we have for the BMS and Parameter Variability models a grid with at least (20 × 20 × 7 × 7 × 7 × 7 × 7 × 7 =) 47,059,600 unique parameter combinations, and for the other models, we have grids with (20 × 7 × 7 × 7 × 7 × 7 × 7 =) 2,352,980 at least parameter combinations.

#### Bayesian Mutation Sampler

The chain length of the BMS is estimated between 6 and 200. This includes the range of chain lengths found for causal reasoning tasks before (Davis & Rehder, 2020). When chain lengths become large, the differences in predictions become negligible (Davis & Rehder, 2020). Therefore, we used equally spaced points between 6 and 200 in terms of their inverse cube root. That is, we computed the inverse cube root (${x}^{-\frac{1}{3}}$) of 6 and 200, then picked 18 equally spaced points between them (so in total, we have 20 values including ${6}^{-\frac{1}{3}}$ and ${200}^{-\frac{1}{3}}$) and then reverted all these points back to the original space and rounding them, resulting in the following sequence of chain lengths: [6, 7, 8, 9, 11, 12, 14, 16, 18, 21, 25, 30, 36, 43, 53, 66, 84, 110, 146, 200].

For the β parameter of the Beta(β, β) prior we wanted the grid values to be picked such that the prior distributions were symmetric around the uniform distribution, which occurs when β = 1. To do this, we first picked values for β < 1 from 0 to 1 with a step size of 0.1. Next, to pick values for β > 1, we computed the total variation distance (Levin & Peres, 2017) between the uniform distribution and each Beta(β, β) distribution with β < 1, and then identified a set of β > 1 that have the same total variation distances from the uniform distribution (Kolvoort et al., 2023). This symmetry about the uniform prior was not used for picking a value symmetric to β = 0, as this would lead to an infinite value, instead we use a value of 100. This procedure gives the following 21 values for β: [0.1, 0.2, 0.3, 0.4, 0.5, 0.6, 0.7, 0.8, 0.9, 1, 1.11, 1.26, 1.45, 1.73, 2.14, 2.83, 4.14, 7.35, 21.54, and 100].

#### Beta Inference Model

**Fig. 6:**
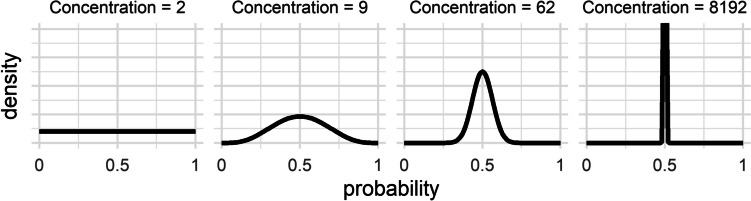
Beta distributions, centered on .5, with a range of concentrations used in the grid search for the Beta Inference model, the Motor Variability model, and the Parameter Uncertainty model. The concentrations plotted here are equally spaced in the set of concentrations used in the grid

The concentration of the Beta distributions predicted as response distributions is the only free parameter for the BIM. The grid values we pick will represent the concentration of the predicted response distribution for the $P\left({X}_{i}=1|Y=1,{X}_{j}=0\right)$ inference, which is the inference which has the lowest concentration (see Table 3). The concentrations of the distributions for the other inference types are determined by multiplying the concentration with the respective concentration scaling factor. At the lower limit of the set of concentrations, we want the model to predict a uniform distribution, while at the upper limit, we want the model to predict no variability, which is for responses to all be at a single percentage point. As the lower limit, we use a concentration of 2, which results in a uniform distribution. Moreover, since for the Beta Inference model the concentration is theoretically equivalent to the number of learning experiences or internally generated samples, 2 is a practical lower limit as a value of 1 would result in only 0% or 100% responses. To determine the upper limit, we identified 2^13 = 8192 to be the lowest power of 2 for which the standard deviation of the Beta distribution would be within half a percentage point, meaning that most of the probability weight would be assigned to a single percentage point. Consequently, the grid range for the concentration parameter is [2^1^, 2^13^]. As with the chain lengths for the BMS, the difference between the predictions becomes smaller for larger concentrations (Fig. 6). Therefore, we applied the same inverse cube root procedure as for the BMS chain lengths, resulting in the following set of concentration values: [2, 3, 4, 5, 6, 7, 9, 11, 13, 17, 23, 30, 43, 62, 96, 158, 290, 620, 1722, 8192].

#### Motor Variability Model

The MVM has concentration as the only free parameter as well. The same reasoning as for the Beta Inference model applies here, so we use the same set of concentration values as for the Beta Inference model.

#### Stimulus Encoding Error Model

For the SEE model, the probability of misreading a stimulus value is the single free parameter. This ranges naturally from 0 to 1, and we picked 19 equally spaced values resulting in the following set of 21 values: [0, 0.05, 0.10, 0.15, 0.20, 0.25, 0.30, 0.35, 0.40, 0.45, 0.50, 0.55, 0.60, 0.65, 0.70, 0.75, 0.80, 0.85, 0.90, 0.95, 1].

#### Parameter Uncertainty Model

The PUM has two free parameters: the concentrations of the Beta distributions from which the causal strengths and base rates are sampled. The mode of these Beta distributions is fixed at the true value of the parameter (0.5 for the causal strengths, 0.5 for the base rate of the cause (Y), and 0.2 for the base rates of the two effects (X_1_ and X_2_), see discussion of the data above). The estimation of the concentration of these distributions is the same for the BIM and MVM models.

#### Guess Model

For the Guess Model, we are restricted to a coarser grid due to computational resource restrictions which stem from the fact that this model is combined with all the aforementioned models. However, by looking at the individual response distributions for each inference type (Fig. 3), we can pick these values in a way that covers plausible values. The first thing to note is that some distributions have all responses at 50% and some have none, so we need to include 0 and 1 as guess proportions. Next, we can observe that in the case of bimodal response distributions, the mode at 50% is never larger than the other mode, meaning that at least half of responses were not at 50%. This leaves the range between 0 and 0.5, and we picked values with step size 0.1 between these limits, resulting in the following set of 7 guess proportions: [0, 0.1, 0.2, 0.3, 0.4, 0.5, 1]. As participants provide 20 responses to each inference type, these parameter values correspond to 0, 2, 4, 8, 10, or 20 guesses respectively.

### Analysis

For our analysis of model predictions and parameters, we will make use of Bayesian Model-Averaging (BMA; Hinne et al., 2020; Hoeting et al., 1999). BMA allows for inference regarding model parameters and predictions while taking into account the uncertainty regarding the best model. It does this by assigning weights to each model based on the posterior model probabilities. These will be computed by comparing the relative Bayesian Information Criterion (BIC) scores (Schwarz, 1978; Wagenmakers & Farrell, 2004). The use of BIC scores and BMA allows us to compare and analyze the generative models even though they differ in the number of free parameters and posterior probabilities. We will use BMA in two ways. First, we will use BMA for each base model to collapse over the guess components. This allows us to analyze and compare the parameters and predictions between base models taking into account the varying guess components. Second, we will use BMA for each possible guess component, meaning that we will collapse across all base models to obtain BMA-weighted estimates of the guess proportions. This will allow us to compare guess components taking into account the varying base models. To compare parameter estimates between groups. we will use the non-parametric Mann–Whitney-Wilcoxon test (Mann & Whitney, 1957; van Doorn et al., 2020) instead of the standard *t*-test, as parameter values are not normally distributed (see the “Experiment” section). We will again use Gini’s Mean Difference (GMD; David, 1968; Yitzhaki, 2003) as an index of the variability of the responses.

## Model Fitting Results

### Overall Model Fit

Our main goal is to identify the most likely theoretical model considering the distributions of causal judgments, and therefore we begin by examining the quantitative fit of each model. To assess relative model fit, we computed BIC weights of all models. We did this at the group and individual levels.

Starting with the group level fits, we find that the BMS is the most likely model Table 5). Aggregating over guess components and computing group-level posterior model weights (Wagenmakers & Farrell, 2004), the BMS is more than 1000 times more likely to be the true data-generating process compared to any of the other models (evidence ratios BMS: 10^293^ versus BIM, 10^120^ versus MVM, 10^57^ versus PUM, and larger than 10^300^ versus SEE). This indicates that the stochastic sampling mechanism underlying the BMS is likely to be an important source of variability in causal judgments.

**Table 5:** Wins, Group BICs, Mean individual BICs, and their standard errors per model. Group BIC refers to group-level BIC values computed using all data. Ind. BIC refers to individual-level BIC values which are averaged over participants to get the mean BIC value and the standard error (in brackets). For the ‘Overall’ row we first selected the best fitting guess component within a base model for each participant and then averaged the BIC values over participants. These overall values indicate the fit of the base models while letting the number of guessing parameters vary per participant. Lower BIC values imply a better fit. The win column refers to the number of participants for which the model is the winning model (out of 29 participants). The guess components are listed per number of parameters, where 1 par refers to a fixed amount of guessing for all inference types, 2 par refers to different guess proportions per reasoning direction, 3 par refers to different guess proportions per type of information provided, and 6 par refers to a different guess proportion per inference type

		BMS			PUM			BIM			MVM			SEE		
		Group BIC	Ind. BIC	Win	Group BIC	Ind. BIC	Win	Group BIC	Ind. BIC	Win	Group BIC	Ind. BIC	Win	Group BIC	Ind. BIC	Win
No guessing		− 3737	− 120.1 (12.8)	10	− 2504	− 77.53 (12.3)	1	− 1154	− 35.40 (17.2)	1	− 2403	− 78.47 (11.5)	3	47,157	1630 (170)	0
Guessing component	1 par	− 4097	− 128.1 (14.3)	1	− 3660	− 113.0 (14.6)	0	− 2540	− 78.78 (20.1)	1	− 3427	− 109.4 (15.0)	0	29,751	1035 (145)	0
	2 par	− 4432	− 135.2 (16.9)	3	− 4049	− 122.0 (17.9)	1	− 2972	− 89.29 (23.3)	0	− 3811	− 118.2 (18.5)	3	29,361	1026 (147)	0
	3 par	− 4277	− 125.5 (15.3)	1	− 3969	− 114.9 (16.0)	0	− 2831	− 80.02 (21.6)	0	− 3676	− 109.2 (16.6)	2	29,471	1034 (145)	0
	6 par	− 4679	− 126.2 (17.8)	2	− 4415	− 117.0 (16.9)	0	− 3327	− 83.92 (24.4)	0	− 4127	− 111.5 (19.7)	0	29,042	1032 (147)	0
Overall			− 143.6 (17.4)	17		− 131.2 (18.6)	2		− 97.84 (24.0)	2		− 127.4 (19.3)	8		1021 (147)	0

We also find that each base model’s group level BIC score is best when a guess component with 6 parameters is added (group BIC columns in Table 5). This result indicates that different guess proportions for each inference type are required to account for the whole dataset.

**Fig. 7:**
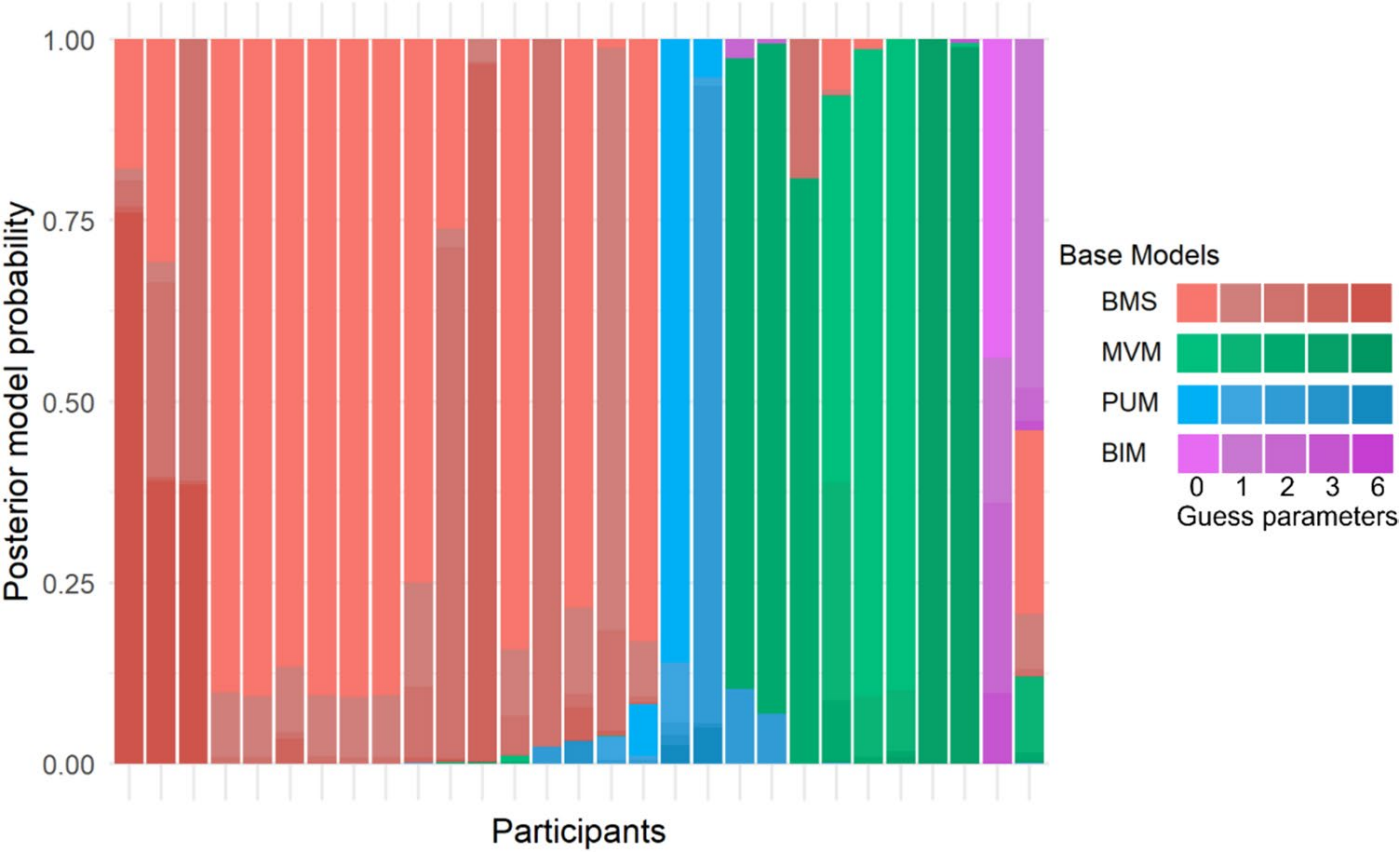
Posterior model probabilities, based on BIC weights, for each participant. Darker colors mean that more guess parameters are estimated for that model. 0 parameter refers to no guessing, 1 parameter to a fixed amount of guessing for all inference types, 2 parameters refers to different guess proportions per reasoning direction, 3 parameters refers to different guess proportions per type of information provided, and 6 parameters refers to a different guess proportion per inference type. The SEE model was excluded from this comparison since the posterior model probability of that particular model was 0 for all participants

Not surprisingly, a more nuanced picture emerges when the models’ performance at the level of individual subject is considered. Considering each base model’s BIC scores averaged over subjects (last row of Table 5), the BMS again emerges as the superior model. In contrast to the group-level results, the individual BIC columns in Table 5 indicate that the most complex 6-parameter guessing model did not always yield the best fit, suggesting that participants may vary in their guess proportions. To explore individual differences in greater detail—asking, for example, whether BMS is the most likely model for every participant—we computed posterior model probabilities (based on BIC weights) for each model separately for each participant (Fig. 7).

The percentage of participants best fit by BMS, MVM, PUM, BIM, and SEE was 59%, 28%, 7%, 3%, and 0%, respectively (Fig. 7). That the majority of participants are best explained by the BMS provides additional evidence suggesting that the sampling scheme of the BMS is an important source of variability. Yet, the fact that a different model—the MVM—yielded the best fit for 28% of participants opens the door to the possibility that the dominant sources of variability can vary per participant. Indeed, Fig. 7 indicates that for each participant except one (the right most column in Fig. 7), there is only one base model that explains their data best.

To address the question why some models (in particular the BMS and MVM) yielded better fits than others, we asked how these models fare at reproducing the relevant behavioral effects identified in the “Experiment” section, including the means and variability by inference types, as well as the more subtle aspects of the response distributions (bi-modal responding and spikes at 50%).

### Predicted Means

We computed the model-averaged predictions for each base model for each participant and inference type. Overall, the differences between observed and predicted means was relatively small, with each model’s predictions being on average around 5 to 6 percentage points off from the observed responses (Table 6). Using repeated-measures ANOVAs with the differences between observed and predicted means as dependent variable and model as independent variable, we find that the models do not differ in terms of how well they predict the mean response (*F*(4, 837) = 0.967, *p* = 0.425, BF_10_ = 0.0061). However, it is important reiterate that means do not provide a good description of the multi-modal response data, and so these results should not be taken to indicate overall model performance (as discussed in the previous section).

**Table 6:** Overall differences between observed and predicted means and GMDs in percentage points. Predictions for each model are obtained by using BMA over the guess components. Standard errors are in brackets

	BMS	PUM	BIM	MVM	SEE
Mean difference	5.481 (0.477)	5.128 (0.344)	6.045 (0.414)	5.344 (0.532)	5.304 (0.420)
GMD difference	5.581 (0.445)	5.880 (0.500)	6.780 (0.555)	5.851 (0.460)	4.804 (0.428)

**Fig. 8:**
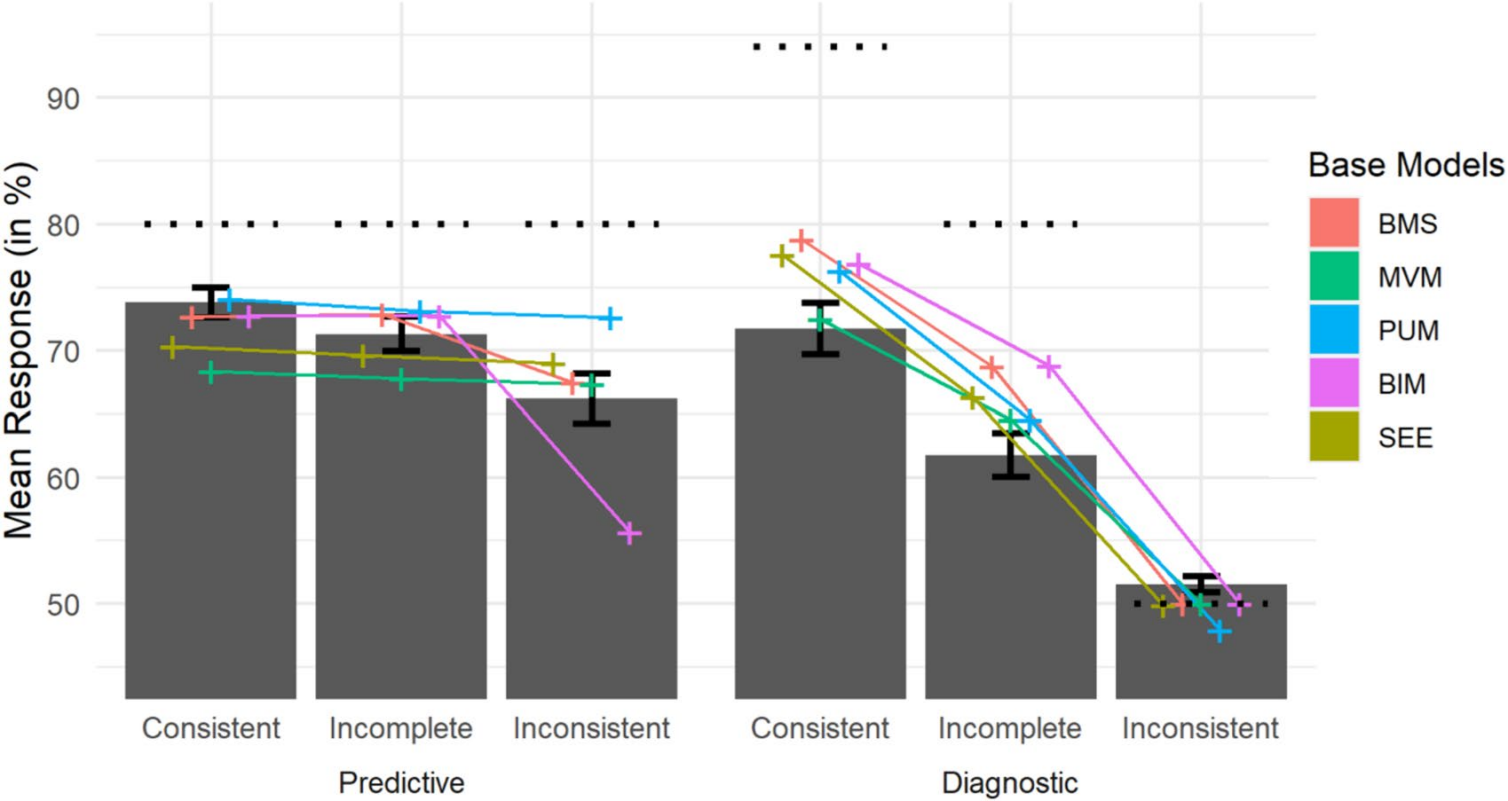
Predicted and observed mean responses per inference type. Bars represent mean responses and error bars their standard error. Crosses indicate mean predictions for each model, these are obtained by using BMA over the Guess Model predictions. Dotted horizontal lines indicate the normative response

Figure 8 indicates how the models fared at reproducing the means for each inference type. From the literature we know that people exhibit mean conservatism (pattern 1a in Table 2) and Markov violations (pattern 2). Figure 8 shows that all models predict mean conservatism, that is, the predictions are between the normative response and 50%. We can see that the BMS most accurately predicts the pattern of Markov violations (Predictive inferences, leftmost three bars in Fig. 8). In contrast, the BIM appears to predict a Markov violation larger than observed and the other models do not appear to predict Markov violations. Separate ANOVAs for each model on the predictive inferences, using Information as independent variable, identify indeed that only for the BMS and BIM there is clear evidence for Markov violations in their predictions (BMS: F(2,56) = 42.7, *p* < 0.001, BF_10_ = 68.3; BIM: F(2,56) = 137.2, *p* < 0.001, BF_10_ > 1000) with evidence for an absence of Markov violations for the other models (MVM: F(2,56) = 3.32, *p* = 0.043, BF_10_ = 0.11; PUM: F(2,56) = 3.02, *p* = 0.056, BF_10_ = 0.13; SEE: F(2,56) = 2.94, *p* = 0.061, BF_10_ = 0.12). For the Diagnostic inferences (rightmost three bars in Fig. 8), we see that the MVM model predictions are closest to the observed means, but all models reproduce the effect of Information. Overall, Fig. 8 indicates that the BMS provides the best account of participants’ means responses.

### Predicted Variability

We also computed the GMD predictions of each base model for each participant (Table 6). Repeated-measures ANOVAs revealed that the models differed in how well they predict GMD (*F*(4, 837) = 4.89, *p* < 0.001, BF_10_ = 2.31). Using post hoc contrasts, we find that this is due to BIM being worse than the SEE (*t*(837) = 4.36, *p* < 0.001) and BMS (*t*(837) = 2.65, *p* = 0.063) models. The fact that the SEE model performs comparably to the other models here, while it is the worst model in terms of posterior model probabilities, is an indication that the data is too complex to be captured by simple indices such as the mean or GMD.

**Fig. 9:**
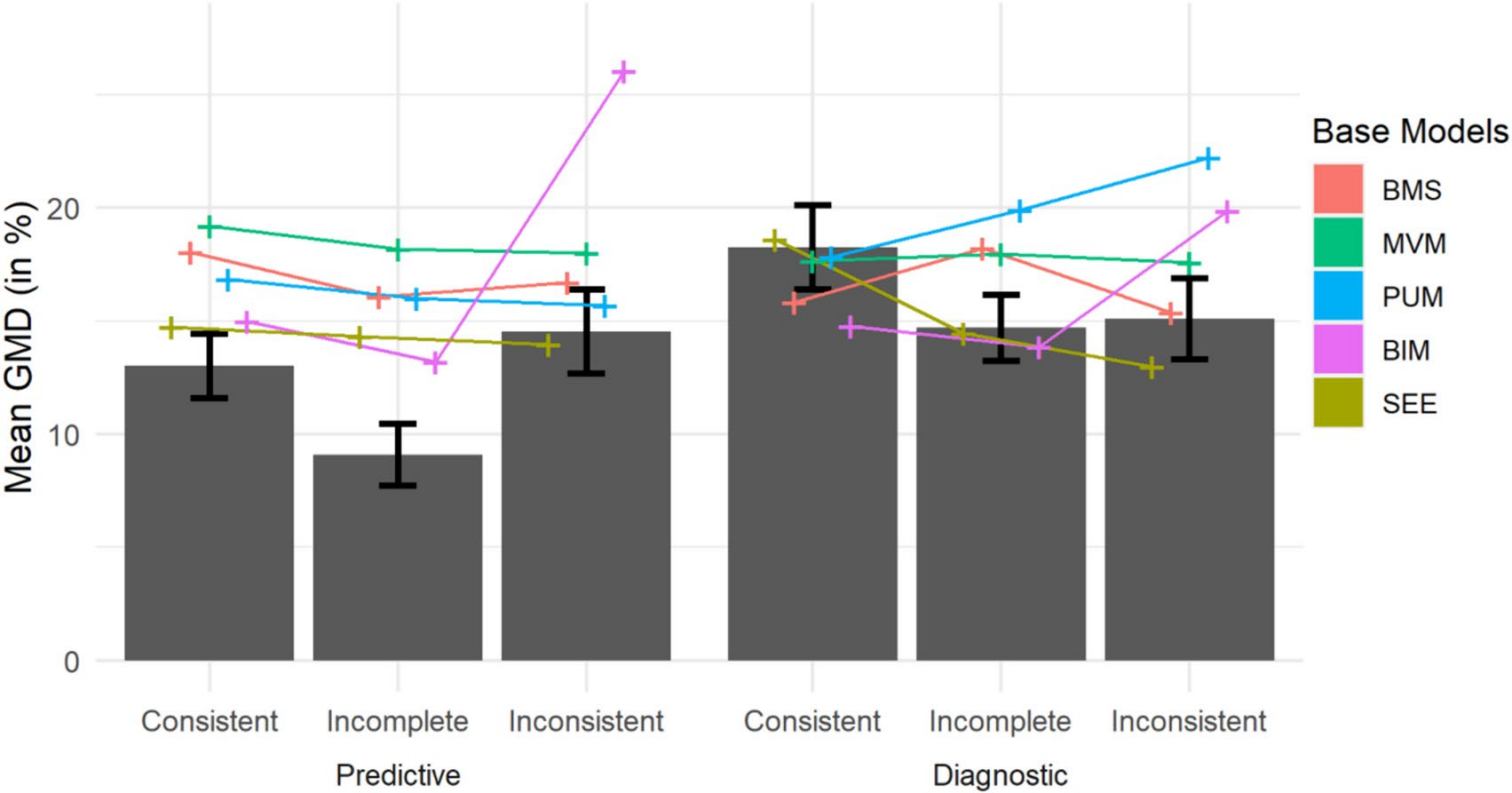
Predicted and observed variability in responses (as indexed by Gini’s Mean Difference, GMD) per inference type. Crosses indicate mean GMD predictions for each model, these are obtained by using BMA over the Guess Model predictions. Bars represent the GMD of responses and error bars their standard error

As we did for the means, we also examined predicted variability (indexed by GMD) for the different inference types. Specifically, we asked whether the models predict that variability is lower for inferences with incomplete information (pattern 3a) and that variability is higher for diagnostic inferences (pattern 3b). We find that only BIM predicts variability to be lowest for inferences with incomplete information (Fig. 9), but only the difference with inconsistent information is significant (*Δ* = 9.34, *t*(812) = 13.2, *p* < 0.001, BF_10_ > 1000), while the difference with consistent information is not (*t*(812) = 2.05, *p* = 0.102, BF_10_ = 0.998). There is evidence that the SEE model predicts GMD for incomplete information to be lower than for consistent information (*Δ* = 2.12, *t*(812) = 2.99, *p* = 0.0081, BF_10_ = 7.68), but it is not different from the incomplete information condition (*t*(812) = 1.03, *p* = 0.313, BF_10_ = 0.238). For the BMS, MVM, and PUM models, there are no significant differences in predicted GMD due to a change in information.

Additionally, only PUM predicts that variability is higher for diagnostic inferences (*Δ* = 3.90, *t*(812) = 6.73, *p* < 0.001, BF_10_ > 1000). No difference between diagnostic and predictive GMD is predicted by BMS (*t*(812) =  − 0.89, *p* = 0.373, BF_10_ = 0.119), MVM (*t*(812) =  − 1.30, *p* = 0.193, BF_10_ = 0.267), and SEE (*t*(812) = 1.72, *p* = 0.086, BF_10_ = 0.486), while BIM predicts that the GMD of predictive inferences is higher (*t*(812) = -3.08, *p* = 0.0021, BF_10_ = 9.45).

Clearly, none of the models adequately captures the observed patterns of within-participant variability as indexed by GMD over inference types in this dataset. Note that as a summary statistic, the GMD is not well suited for capturing all (qualitative) aspects of participant’s response distributions. This is evidenced by the fact that the SEE model performs comparable to other models in predicting GMD while being clearly outperformed in terms of quantitative fit (“Overall Model Fit” section). Accordingly, we now examine how well the models reproduced those response distributions.

### Predicted Distributions

**Fig. 10:**
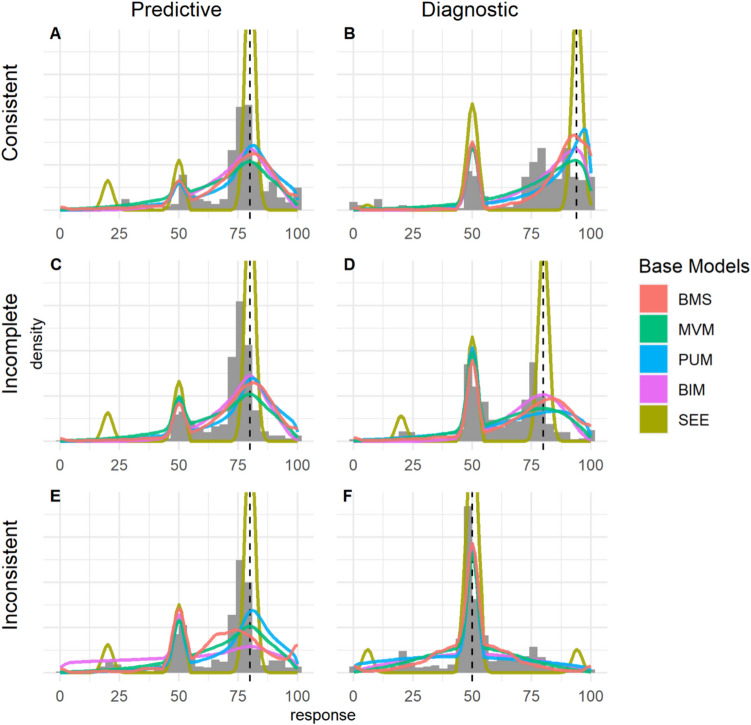
Predicted and observed response distributions per inference type. The grey histograms represent participant responses. The colored lines are the predictions for each base model. These predictions are obtained by model averaging over the Guess Model predictions. Dotted vertical lines indicate the normative response. Because the SEE model predicts substantially more probability mass close to the normative response, the y-axis has been cut-off to allow for better comparison of the densities of the other models

The model weighted response distributions of each base model together with the observed distributions are presented in Fig. 10. Note that because the predicted distributions are fitted with a single set of parameters, often only 1 or 2, for all 6 inference types, an exact fit to the shape of the distribution cannot be expected. However, the successful models should be able to capture the qualitative patterns in responses identified in the introduction.

We now separately discuss how the predicted distribution of each fitted base model matches the empirical distributions. Nevertheless, note that there are some properties of the fitted distributions that hold for all models. First, all models correctly predict multimodal response distributions (pattern 4). All predict two modes (except for the SEE model, which predicts three), with one mode near 50% (pattern 5a) and another near the normative response. Moreover, all models correctly predict the mode at 50% is larger for inconsistent versus incomplete versus consistent inferences (pattern 5b) and for diagnostic versus predictive inferences (pattern 5c).

#### SEE Model

The fit of the SEE model to the empirical distributions is presented in Fig. 10. Apart from the successes mentioned above, the SEE model fails to reproduce an important qualitative feature of the empirical distributions. In particular, in the empirical distributions the bulk of responses are conservative, that is, fall between the normative response and 50% (pattern 1b), which is not predicted by the SEE model. Interestingly, SEE predicts a third mode of responses below 50%, and in fact Fig. 10 reveals there are very small clusters of responses near 25% for some inferences (Fig. 10A, D, E, and F). This suggests that participants are occasionally misreading stimulus values, although the exact position of these clusters are not always where SEE predicts (Fig. 10A and F), this mismatch might be due to participants rounding their responses. We return to this observation in the “Discussion” section.

#### PUM Model

Figure 10 reveals that, like the SEE, the PUM model fails to predict that the bulk of responses fall between the normative response and 50%. Instead, for predictive inferences (Fig. 10A, C, and E) the prediction is that the distribution of responses near the normative response are right-skewed. For the diagnostic inferences in Fig. 10B and D, the right-most mode is greater than the normative response, unlike in the empirical distribution. In other words, the PUM predicts too many anti-conservative responses, that is, it fails to adequately reproduce the lack of extreme responses (pattern 1c).

#### BIM Model

Figure 10 reveals that the BIM model correctly predicts responses between the normative response and 50%, although not for the predictive inconsistent inferences, where it predicts a distribution near the normative response that is too flat (Fig. 10E). Note that even when the BIM predicts conservative responses overall, it continues to overpredict the number of anti-conservative responses (e.g., Fig. 10C and D).

#### MVM Model

Figure 10 reveals that the MVM model correctly predicts conservative responses between the normative response and 50% even for the predictive inconsistent inferences (unlike the BIM). Nevertheless, although it reproduces moderate conservatism, it tends to, overestimate anti-conservative responses, similar to PUM and BIM (e.g., Fig. 10C, D, and E).

#### BMS Model

The fitted distributions of the BMS model (Fig. 10) are qualitatively very similar to those of the MVM. One difference is that for the predictive inconsistent inference, it correctly predicts that the mode near the normative response is conservative, but it predicts more conservative responses than observed (Fig. 10E). In addition, for some inferences, the number of extreme, anti-conservative responses incorrectly predicted by the BMS is larger than those predicted by the MVM (e.g., Fig. 10B, D, and E).

Taken together, the BMS and MVM perform best in terms of predicting these qualitative patterns in the distributions. While neither capture the distributions perfectly, the other models fare worse. This is in line with the posterior model weights (Fig. 7), which indicate that the BMS is the most likely model for the majority of participants, followed by a sizeable group best fit by the MVM. This indicates that the dominant source of variability is different for these two groups of participants, possibly due to using different strategies.

**Fig. 11:**
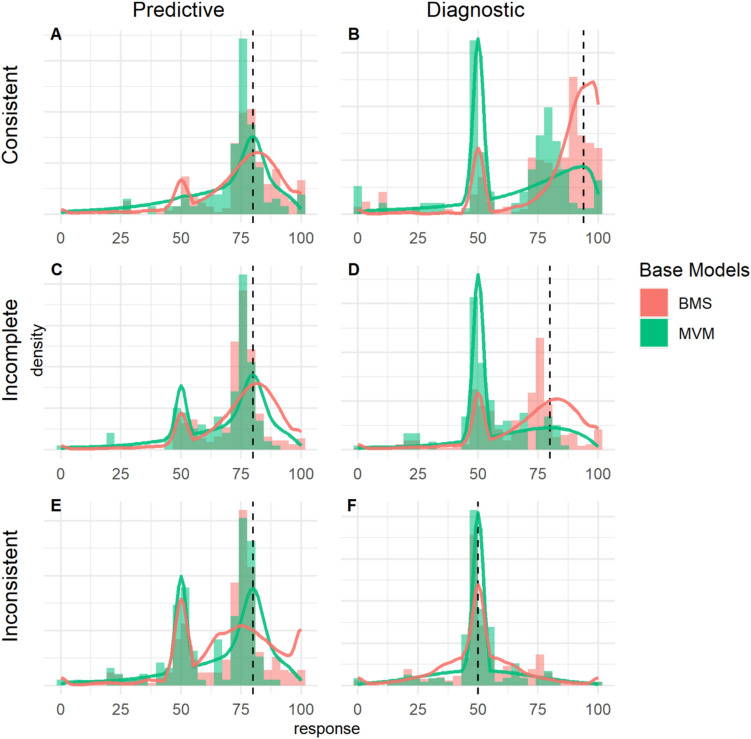
Predicted and observed response distributions per inference type separated for the participants that were best fit by either the BMS or MVM model (25 out of 29 participants). The red and green histograms represent the responses of participants best fit by the BMS and MVM models respectively. The red and green lines represent the model predictions for those groups of participants of the BMS and MVM models respectively. These predictions are obtained by model averaging over the Guess Model predictions. Dotted vertical lines indicate the normative response

A question that arises from this is what differentiates these two participant groups in terms of their behavior. To investigate this, we separately plotted the distributions of responses of these two groups together with their best fitting model predictions (Fig. 11).

One property that distinguishes these groups is that participants best fit by the MVM model are more conservative than those best fit by the BMS: the mode at 50% is higher, the second mode is closer to 50%, and there are fewer extreme responses, especially for the diagnostic consistent and incomplete inferences (Fig. 11B and D). However, although the MVM participants were more conservative and the BMS participants less so, we do not believe that these are intrinsic properties of the models, as the β prior parameter of the BMS can in principle yield any level of conservatism. That is, it is not conservatism that makes the MVM fit better for the MVM participants than the BMS, but rather that the variability of their responses is more characteristic of motor variability.

While we believe that the BMS and MVM provide reasonable first-order approximations to the observed distributions, we acknowledge the presence of some qualitative mispredictions. Both models overpredict the number of anti-conservative responses. In particular, whereas their modal responses tend to be at or above the normative response (except for the BMS prediction for predictive inconsistent inferences), participants’ modal response tends to be below the normative response. This is especially apparent for the diagnostic consistent and incomplete inferences (Fig. 11B and D). That is, whereas both models predict conservatism overall, they are not as conservative as participants. In the “Discussion” section, we will discuss whether participants’ rounding of responses might be one factor responsible for this misprediction.

### Fitted Parameter Values

To validate the model fits and further investigate the differences between groups of participants, we also looked at the mean fitted parameter values for each model (Table 7). For the BMS, we find that the average chain length is within the range expected from the literature, albeit on the higher side (Davis & Rehder, 2020; Kolvoort et al., 2023; Zhu et al., 2020). For β, we find a similar value as in a previous study (Kolvoort et al., 2023), suggesting that participants used a prior distribution of a shape close to the uniform distribution (β = 1), which reflects relatively weak conservatism. This finding is consistent with the lower rate of conservative responses predicted by the BMS fits (Fig. 10).

**Table 7:** Estimated mean parameters of base models for all participants. These parameter values are model-averaged using BMA over the Guess Model predictions. ‘Concentration’ is abbreviated as ‘Conc.’

	BMS		PUM		MVM	BIM	SEE
Parameter	Chain length	β	Conc. causal strengths	Conc. base rates	Conc.	Conc.	Error prob.
Mean	70.30	1.218	29.74	347.5	15.07	5.711	0.1081
SD	54.8	1.32	62.6	1542	32.3	12.1	0.0854

The parameters of the models that fit poorly are also of some interest. The SEE model estimates the probability of misreading stimulus values to be on average 11%, a result that potentially accounts for the small cluster of responses observed at 25%. Of course, this estimate should not be considered highly reliable given the poor fit of the SEE model. For the PUM, we find that the uncertainty was a lot higher for base rates than it was for the causal strengths. Base rates have a larger influence on diagnostic inferences, as CBN theory stipulates that diagnostic reasoning requires the incorporation of the base rate probability of the cause. So, more uncertainty in base rates leads to more variable diagnostic inferences compared to predictive ones, which is what we observe in participants (pattern 3b) and the PUM predictions (Fig. 9). This is also in line with previously argued claims that diagnostic inferences tend to be more difficult (Fernbach & Darlow, 2010; Fernbach et al., 2011). We find that the concentrations for the BIM model are lower than for the MVM, which is also expected as the BIM stipulates the concentration to be higher for certain inferences than the fitted parameter (Table 3). Overall, these parameter values are consistent with the observed responses, which suggests that the models were implemented and fit correctly.

### Guess Proportions

Lastly, we conducted an exploratory analysis of the Guess Model and the estimated guess proportions. While the Guess Model was not of primary interest, we provide a short analysis as it can shed light on the necessity of implementing such a mechanism. We found that for 14 out of the 29 participants, the best fitting model included at least one guess parameter (see shading in Fig. 7). This is evidence for the idea that the observed distributions of responses are due to multiple processes: a reasoning process and guessing. Moreover, multiple of the participants best fit by the BMS had nonzero guess parameters (Fig. 7), implying that the default response mechanism of the BMS cannot by itself account for the large spikes of responses at 50%.

**Fig. 12:**
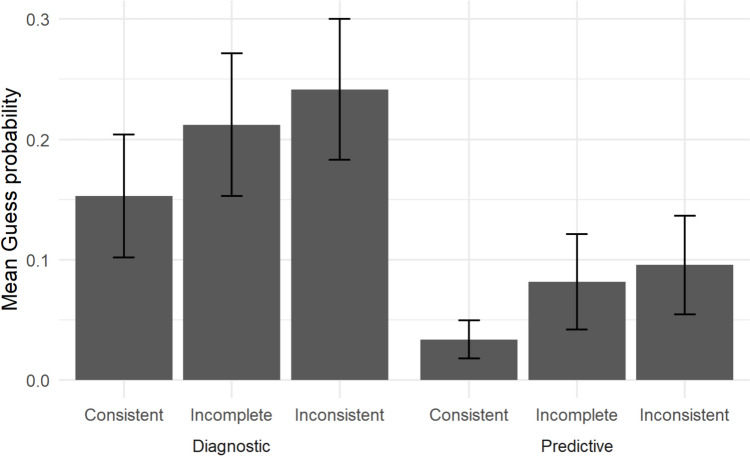
Bayesian Model-Averaged estimates of the probability of guessing for each inference type. Error bars indicate the standard error

To investigate whether guessing was affected by the type of inference, we computed model-weighted guess proportions for each participant (Fig. 12). We find an overall mean guess probability of 0.136 (*SE* = 0.035) which is significantly above zero (*t*(28) = 3.94, *p* < 0.001, *BF*_*10*_ = 63.1) indicating that indeed participants guessed on a sizable number of trials. To test whether the direction of reasoning or the provided information affected the amount of guessing, we use a repeated measures ANOVA. There was a significant effect of reasoning direction (*F*(1, 140) = 22.0, *p* < 0.001, BF_10_ = 38.9), with participants guessing more on diagnostic trials (*M* = 0.202, *SE* = 0.053) than on predictive trials (*M* = 0.0703, *SE* = 0.029). This finding squares with previously reported claims that diagnostic inferences are experienced as more difficult (Fernbach & Darlow, 2010; Fernbach et al., 2011). Although Fig. 12 seems consistent with our prior expectation that more inconsistent information would lead to more guessing, we observed some evidence against this effect (*F*(2, 140) = 2.53, *p* = 0.083, BF_10_ = 0.179).

## Discussion

This study had two major aims. The first was to use a novel repeated-measures design that could establish the existence of meaningful within-participant variability, that is, variability related to the process responsible for generating causal judgments. Having established such within-participant variability, our second goal was to disambiguate between theoretical accounts of causal reasoning. This was possible by capitalizing on our novel experimental setup and on the ability of relevant theoretical proposals to account for the within-participant variability. The following section reviews our new empirical findings and the implications those findings have for models of causal reasoning. We close with a discussion of limitations of this work and suggestions for future research.

### Empirical Findings

A main challenge for computational models that strive to explain variability in human performance in any task is to clearly distinguish between variability that arises from within- versus between-participant sources. Doing so is important because the cognitive mechanisms responsible for these sources can differ dramatically. Yet we also noted that a main experimental challenge to establishing the existence of meaningful within-subject variability in causal reasoning is obtaining independent measures, because, for example, a reasoner answering the same causal query repeatedly may feel obligated to respond with the same answer (thus yielding an estimate of variability that is too low). We addressed this problem by presenting several variants of the same logically-equivalent causal inference, variants with surface properties that were unlikely to remind the reasoner of previous presentations. Specifically, the same logical inference was asked in different content domains, involving different variables that nevertheless played a symmetric role in the causal network, and asked participants to estimate either the presence and absence of a variable. This procedure resulted in each participants generating 20 estimates for the same inference, allowing us to study within-subject variability in causal reasoning.

This experiment established variability as a property of the responses of a single individual presented with multiple instances of the same causal inference. Moreover, it found that variability exhibits a number of important qualitative features. The magnitude of within-participant variability was lower for inferences with incomplete versus complete information and higher for diagnostic versus predictive inferences. The response distributions were typically multimodal, often with one mode at 50%, and the tendency to respond at 50% seemed larger for diagnostic inferences vs. predictive ones and for inconsistent inferences vs. consistent ones. Although variability in causal reasoning has been reported previously (Kolvoort et al., 2024; Rehder, 2018; Rottman & Hastie, 2016), here we show that it can be used to distinguish between generative models of causal judgments.

We acknowledge that our repeated-measures methodology may not be perfect. For example, it is conceivable that participants reason differently in different domains, perhaps because they assume different causal model parameters (causal strengths, variable base rates) in each domain. Within each domain, it is also possible that participants would reason about one of the effect variables (in the domain of economics, a high level of retirement savings) differently than the other (small trade deficits). We attempted to eliminate such differences by specifying causal model parameters that were the same across domain and effect variables, but of course we cannot be certain that all participants fully incorporated these parameters into their mental causal model. Nevertheless, post-hoc analyses revealing the absence of differences in participants’ causal inferences across content domains corroborates our assumption that inferences from the different domains could be treated equivalently. Overall, we believe that we largely succeeded in obtaining independent repeated measures and so provide a veridical estimate of within-participant response variability.

### Model Evaluation

The BMS had by far the best quantitative fit to the data, being at least 1000 times more likely to account for the data than any of the other models. Recall that we considered numerous sources of within-participant response variability, including that participants sometimes misread the stimulus values (as codified by the SEE model), varied in the causal model parameters they used from trial to trial (PUM) or that variability arose from motor responses or general task noise (MVM). We also considered a model—the BIM—that, like the BMS, posits that variability emerges from stochastically sampling over the state space of the causal network. That the BMS emerged so decisively as the best model provides evidence that the specific stochastic sampling process it posits—MCMC sampling over causal states with biased starting points—is the dominant mechanism responsible for variability in causal reasoning.

This strong conclusion in favor of the BMS was somewhat moderated when the fits to individual participants were considered. Whereas the majority (59%) of participants were best fit by the BMS, a substantial minority (28%) were best fit by the MVM. Notably, every participant except one had a clear winning model, suggesting that people likely differ in terms of what process is the dominant source of their variability.

Despite the strong success of the BMS and to some extent the MVM, it was clear that neither they nor any of the other models captured all the qualitative empirical patterns. Table 8 summarizes which of the main empirical phenomena originally presented in Table 2 are accounted for by each of the models we tested.

**Table 8:** Qualitative patterns and model predictions

Qualitative patterns	Predictions				
	BMS	MVM	PUM	BIM	SEE
1a. Mean conservatism	✓	✓	✓	✓	✓
1b. ‘moderate’ conservatism	✓	✓	X	✓, but not for all	X
1c. Extreme responses are rare (mostly just for consistent inferences)	✓, except for two inferences	✓	✓	✓	✓
2. Markov violations	✓	X	X	✓, but too strong	X
3a. Within-participant variability is lower for incomplete information	X	X	X	✓	X
3b. Within-participant variability is higher for diagnostic inferences	X	X	✓	X	X
4. Multi-modal response distributions	✓	✓, by guesses	✓, by guesses	✓, by guesses	✓, 3 modes
5a. Spikes at 50%	✓	✓, by guesses	✓, by guesses	✓, by guesses	✓
5b. Spikes at 50% increase with inconsistency of information provided	✓	✓, by guesses	✓, by guesses	✓, by guesses	X, not strictly for diagnostic inferences
5c. spikes at 50% are larger for diagnostic inferences	✓	✓, by guesses	✓, by guesses	✓, by guesses	✓

One of the important features of causal reasoning we aimed to account for is conservatism, the tendency of people to shift their response towards the middle of a response scale. Whereas all models predicted mean conservatism (pattern 1a) and usually the lack of extreme responses (pattern 1c), only the BMS and MVM predicted the more subtle property that the bulk of the responses fell between the normative response and 50% (pattern 1b). Yet, comparison of the response distributions of these models with participants’ indicated that the models are not sufficiently conservative, as revealed, for example, by the fact that their modal responses were at or above the normative response whereas those of participants were below it (Fig. 10).

Another interesting aspect of the response distributions is the presence of small clusters of responses at 25%, clusters whose presence the SEE predicts but not always exactly at 25%. One potential explanation for these mis-predictions is that participants “rounded” their responses, that is, they tended to funnel their responses into one of three response categories centered at 0.25, 0.50, or 0.75. For example, although the normative response to diagnostic consistent inferences was 0.94, examination of participants’ response distributions for this inference (Fig. 10B) reveals modes just below the normative response (perhaps reflecting conservatism) and another near 0.75 (perhaps reflecting rounding). Previous work involving probability judgments have also suggested the possible involvement of rounding (e.g. Costello & Watts, 2014; Kleinjans & van Soest, 2014; Wallsten et al., 1993). We recommend that future research should aim to account for the location of modal responses, perhaps by incorporating rounding processes. Of course, a call for models to account for response means and modes as well as the bulk of the response distributions is another reminder that researchers should aim to account for entire response distributions.

The models were also unable to account for how within-participant variability varied over the six inference types (patterns 3a and 3b). However, these patterns should perhaps be considered preliminary until they are replicated, because they emerged as a result of our novel repeated measures experimental design. But should they prove to be robust, we suspect that accounting for them will require allowing model parameters to vary over the inference types. For example, for the BMS one might assume that reasoners adopt a longer chain length (i.e. sample more) when presented with a seemingly challenging inference in order to improve their accuracy (Zhu et al., 2020). One might consider allowing the parameters of the other models to vary (e.g., the probability of misreading the stimulus, the uncertainty regarding underlying causal parameters, etc.) although providing a psychological justification for why they would vary with inference type might be challenging.

Finally, a third important response pattern is that spikes of responses are present at 50%. Of all the base models, we considered only the BMS and the SEE models naturally predict these spikes, although all models did so when combined with one or more guessing parameters. When considering group level fits, all models—even the BMS and SEE—yielded the best fit in the presence of guessing parameters. Moreover, when individual fits were considered, a substantial minority of participants best fit by the two winning models—the BMS and MVM—were fit best with one or more guessing parameters. These results suggest that the spikes at 50% are partly the result of guessing, a hypothesis suggested by multiple authors (Kolvoort et al., 2023, 2024; Rottman & Hastie, 2016). While the phenomenon of guessing may not be the primary focus of researchers investigating causal cognition, it is nevertheless an important factor that needs to be considered. For example, our results demonstrated that participants are more likely to guess on certain types of inferences than others, which could introduce bias when analyzing differences in mean responses between inference types. Therefore, it is important to take the effects of guessing or default responding into account when interpreting results and drawing conclusions from causal reasoning experiments.

Although they were not the main focus of this article, another important feature of these data were Markov violations (pattern 2). The BMS accurately predicted Markov violations, which is a significant advantage of this model over its competitors, given that such violations are a hallmark feature of human causal reasoning (Ali et al., 2011; Davis & Rehder, 2020; Mayrhofer & Waldmann, 2015; Park & Sloman, 2013, 2014; Rehder, 2014, 2018; Rehder & Waldmann, 2017; Rottman & Hastie, 2014, 2016; Sloman & Lagnado, 2015; M R Waldmann et al., 2008). Although the BIM also predicts the occurrence of Markov violations, it substantially overestimated their magnitude. Of course, it was expected that both models would predict Markov violations, given that both were explicitly designed to do so (Davis & Rehder, 2020; Rottman & Hastie, 2016). However, the BIM’s significant mis-prediction of Markov violations here provides additional evidence that its sampling procedure is not an accurate model of how humans generate causal judgments (and why they commit Markov violations).

In summary, taking together both the quantitative fit to the data and the models’ ability to predict qualitative patterns of interest (Palminteri et al., 2017), we find that the BMS outperforms the other models, suggesting that the stochastic sampling process it embodies is a good candidate for how people generate causal judgments. However, that the BMS alone cannot explain all facets of the data suggests that doing so may require combining it with other models. We found that a sizeable minority of participants were best fit by variants of BMS that incorporated guessing. It might also be necessary to combine the BMS with a rounding process to account for the cluster of responses at 75%. And, it might be necessary to assume both rounding and that stimulus values are occasionally misread to account for the small cluster of responses at 25%. Lastly, even though the BMS outperforms the MVM, it is reasonable to assume that motor noise plays some role even if causal judgments are generated using the sampling procedure posited by the BMS, and so it might be necessary to combine the BMS and MVM to capture the behavior of all participants.

### Limitations and Future Research

The most obvious limitation regarding our conclusions regarding within-subject variability is that they are largely based on a single experiment, the results of our novel repeated measures experimental design. Although our findings show that it is possible to model the distribution of raw responses (see also Kolvoort et al., 2023), more repeated-measures studies are needed to validate the patterns of within-participant variability we observed. This would also allow to determine if those patterns generalize to alternative experimental conditions, and how they can be best accounted for in terms of competing generative models. For example, because the current study was limited to only six inference types and a single causal structure (a three-variable common cause network) with one set of causal strengths and base rates, future research should strive to characterize within-subject variability for other inference types and causal networks. Similarly, while the materials we used have been validated in previous studies (Rehder, 2014, 2018; Rehder & Hastie, 2001; Rehder & Waldmann, 2017), the generalizability of our findings should be tested using a variety of materials (e.g. using different descriptions of causal networks, variables, and relationships). Moreover, because causal cognition underpins a myriad of judgments in other domains, such as categorization, moral judgments, interventions, and learning (Sloman & Lagnado, 2015), efforts should be made to assess whether our conclusions generalize to these other judgment types as well. Indeed, the Mutation Sampler, the model of which the BMS is a generalization, has already been shown to extend to categorization and intervention studies (Davis & Rehder, 2020), and we assume this property is inherited by the BMS.

Another type of important generalization relates to response scales, the format in which participants are asked to provide a causal judgment. We and others (e.g., O’Neill et al., 2022) have argued that people’s causal beliefs are graded (and all studies using a Likert scale, probability, or percentage format implicitly endorse this position), but the mapping of such graded causal beliefs to gradation on a response scale is unlikely to be one-to-one. Exactly how beliefs map onto a probability (or percentage) scale is still an open question. Some studies have partially avoided this issue by fitting a scaling parameter to map responses to a 100-point probability scale (e.g. Davis & Rehder, 2020) or using alternative response formats such as frequency (‘the number of instances out of 20’, e.g. Rottman & Hastie, 2016), Likert scales (e.g. Icard et al., 2017), or having participants choose the most likely causal network state out of two states (e.g. Rehder, 2014). However, none of these options completely avoid the issue of response mapping. One way forward would be to test competing theories on multiple response formats that, while labor intensive, could provide valuable evidence for competing explanations that is to some degree independent of response format.

The aforementioned experimental extensions would allow the candidate theories to be tested on a richer and more varied set of data, allowing for stronger inferences. Both the current study and a previous one we conducted (Kolvoort et al., 2023) focused on full response distributions and we believe that the fact that those analyses were able to so clearly identify a winning model adds credence to the view that variability in causal judgments can be used disambiguate between theoretical accounts of causal reasoning. Other studies have focused on additional dependent variables, including response times (Kolvoort et al., 2024; Rehder, 2014) and confidence judgments (Kolvoort et al., 2024; O’Neill et al., 2022). We believe that the BMS is well positioned to potentially account for these other measures as well (e.g., its chain length parameter can be directly related to response times, Kolvoort et al., 2023). But more generally, we view the use of a broader set of dependent variables as an important positive development for the field.

Incorporating more data, however, comes with additional modeling challenges. The simulation-based modeling approach we developed and used here can be extended to incorporate more data sources as well as more (combinations of) models, including ones without a known analytic form. In the current study we were limited by computational resources, which prohibited the inclusion of more parameter values as well as more combinations of models. Future increases in computational resources will help with this and so will new developments in model fitting and evaluation. One promising technique for studying generative models is that of using amortized inference combined with deep neural networks (Fengler & Frank, 2020; Radev et al., 2020, 2022). Amortized inference refers to the process of separating inference and training such that the inference costs are minimized. We applied such a technique here by first constructing a pre-paid grid with all the model predictions (Mestdagh et al., 2019), after which computing maximum likelihoods (i.e. inference) was very fast. Amortized inference allows for re-use, i.e. other researchers can use our grid to fit the candidate models here to datasets from similar experiments. Instead of simulating a grid filled with model predictions, recent approaches train a neural network to learn the mapping between model parameters and predictions. Implementations of this method are now becoming available (e.g. the BayesFlow package for R; Radev et al., 2022) and will allow researchers to pool computational resources to fit a variety of generative models.

## Conclusion

Past research into causal reasoning has shown that a variety of computational models can account for different patterns in average causal judgments (e.g. Mistry et al., 2018; Rehder, 2014, 2018; Rottman & Hastie, 2016). There are numerous combinations of these models that could account for all the patterns in average judgments, which puts the field in position from which it is hard to come to a satisfactorily account of causal reasoning as there are too many possible model combinations (Rottman & Hastie, 2016). The current research aimed to make progress on this problem by looking at whether we can use the variability in causal judgment to disambiguate between theoretical accounts of causal reasoning and identify the source of this variability. That is, we considered the variability in causal judgments as something to be explained and not to be averaged out.

Our findings suggest that the sampling procedure proposed by the BMS is a substantial source of variability in probabilistic causal judgments. In addition, our analysis indicates it is important to incorporate ‘non-reasoning’ processes into models of causal reasoning, such as guessing and rounding, to improve the ability to capture human response data.

Overall, our study highlights the potential of computational modeling to illuminate the underlying mechanisms of human causal reasoning, and points to new avenues for experimental and modeling research that could lead to a more comprehensive understanding of this form of cognition.

## Supplementary Information

Below is the link to the electronic supplementary material.

Supplementary file1 (DOCX 235 KB)

## Data Availability

The data is publicly available at https://osf.io/dpwg6/

## Footnotes

1 Also known as causal graphical models.

2 Due to an error in the materials, three diagnostic trials were removed from the Economics domain for all participants, resulting in 19 judgments for the diagnostic inferences.

3 If, instead, we would assume that people generate an equal number of samples for each inference then the model would be equivalent to the Motor variability model discussed below.

4 We use the term ‘base rate’ to refer to the probability of a causal variable being present without its causes in the modeled causal structure being present. This is sometimes referred to as the “strength of alternative causes”.

5 For the β prior parameter of the BMS and for the probability of misreading a stimulus value *m* of the SEE model, we use 21 values as they naturally lend themselves to picking an odd number of values, see below.

## Code Availability

Code is available on request to the authors.

## References

[CR1] Ali, N., Chater, N., & Oaksford, M. (2011). The mental representation of causal conditional reasoning: Mental models or causal models. *Cognition,**119*(3), 403–418. 10.1016/j.cognition.2011.02.005 21392739 10.1016/j.cognition.2011.02.005

[CR2] Bogacz, R., Wagenmakers, E. J., Forstmann, B. U., & Nieuwenhuis, S. (2010). The neural basis of the speed-accuracy tradeoff. *Trends in Neurosciences,**33*(1), 10–16. 10.1016/j.tins.2009.09.002 19819033 10.1016/j.tins.2009.09.002

[CR3] Costello, F., & Watts, P. (2014). Surprisingly rational: Probability theory plus noise explains biases in judgment. *Psychological Review,**121*(3), 463–480. 10.1037/a0037010 25090427 10.1037/a0037010

[CR4] Danks, D. (2014). *Unifying the mind: Cognitive representations as graphical models*. MIT Press.

[CR5] Dasgupta, I., & Gershman, S. J. (2021). Memory as a computational resource. *Trends in Cognitive Sciences,**25*(3), 240–251. 33454217 10.1016/j.tics.2020.12.008

[CR6] David, H. A. (1968). Gini’s mean difference rediscovered. *Biometrika,**55*(3), 573–575.

[CR7] Davis, Z. J., & Rehder, B. (2017). The causal sampler: A sampling approach to causal representation, reasoning and learning. *CogSci,**2017*, 1–6. 10.1002/jgrd.50244

[CR8] Davis, Z. J., & Rehder, B. (2020). A process model of causal reasoning. *Cognitive Science,**44*(5), 1–41. 10.1111/cogs.12839 10.1111/cogs.12839 32419205

[CR9] Fengler, A., & Frank, M. J. (2020). Encoder-decoder neural architectures for fast amortized inference of cognitive process models. In S. Denison, M. Mack, Y. Xu, & B. Armstrong (Eds.), *Proceedings of the Annual Meeting of the Cognitive Science Society *(Vol. 42), (pp. 1859–1865). Cognitive Science Society.

[CR10] Fernbach, P. M., & Darlow, A. (2010). Causal conditional reasoning and conditional likelihood. *Proceedings of the 32nd Annual Conference of the Cognitive Science Society,**1*, 1088–1093.

[CR11] Fernbach, P. M., Darlow, A., & Sloman, S. A. (2011). Asymmetries in predictive and diagnostic reasoning. *Journal of Experimental Psychology: General,**140*(2), 168–185. 10.1037/a0022100 21219081 10.1037/a0022100

[CR12] Hastings, W. K. (1970). Monte Carlo sampling methods using Markov chains and their applications. *Biometrika,**57*(1), 97–109.

[CR13] Hinne, M., Gronau, Q. F., van den Bergh, D., & Wagenmakers, E. J. (2020). A conceptual introduction to bayesian model averaging. *Advances in Methods and Practices in Psychological Science,**3*(2), 200–215. 10.1177/2515245919898657

[CR14] Hoeting, J. A., Madigan, D., Raftery, A. E., & Volinsky, C. T. (1999). Bayesian model averaging: A tutorial. *Statistical Science,**14*(4), 382–401. 10.1214/ss/1009212519

[CR15] Holmes, W. R. (2015). A practical guide to the Probability Density Approximation (PDA) with improved implementation and error characterization. *Journal of Mathematical Psychology,**68–69*, 13–24. 10.1016/j.jmp.2015.08.006

[CR16] Icard, T. F., Kominsky, J. F., & Knobe, J. (2017). Normality and actual causal strength. *Cognition,**161*, 80–93. 10.1016/j.cognition.2017.01.010 28157584 10.1016/j.cognition.2017.01.010

[CR17] Johnson, N. L., Kotz, S., & Balakrishnan, N. (1995). *Continuous univariate distributions,* (Vol. 2). John Wiley & Sons.

[CR18] Jones, M. C., Marron, J. S., & Sheather, S. J. (1996). A brief survey of bandwidth selection for density estimation. *Journal of the American Statistical Association,**91*(433), 401–407.

[CR19] Katsimpokis, D., Hawkins, G. E., & van Maanen, L. (2020). Not all speed-accuracy trade-off manipulations have the same psychological effect. *Computational Brain and Behavior,**3*(3), 252–268. 10.1007/s42113-020-00074-y

[CR20] Kleinjans, K. J., & van Soest, A. (2014). Rounding, focal point answers, and nonresponse to subjective probability questions. *Journal of Applied Econometrics,**29*, 567–585. 10.1002/jae

[CR21] Kolvoort, I. R., Temme, N., & Van Maanen, L. (2023). The Bayesian Mutation Sampler explains distributions of causal judgments. *Open Mind,**7*, 318–349. 10.1162/opmi_a_00080 37416078 10.1162/opmi_a_00080 PMC10320818

[CR22] Kolvoort, I. R., Fisher, E., Van Rooij, R., Schulz, K., & Van Maanen, L. (2024). Probabilistic causal reasoning under time pressure. *PLoS ONE,**19*(4), e0297011. 10.1371/journal.pone.0297011 38603716 10.1371/journal.pone.0297011 PMC11008876

[CR23] Levin, D. A., & Peres, Y. (2017). *Markov chains and mixing times*. American Mathematical Society.

[CR24] Lin, Y.-S., Heathcote, A., & Holmes, W. R. (2019). Parallel probability density approximation. *Behavior Research Methods,**51*, 2777–2799. 10.3758/s13428-018-1153-1 31471826 10.3758/s13428-018-1153-1

[CR25] Maaß, S. C., De Jong, J., Van Maanen, L., & Van Rijn, H. (2021). Conceptually plausible Bayesian inference in interval timing. *Royal Society Open Science,**8*(8), 201844. 10.1098/RSOS.201844 34457319 10.1098/rsos.201844 PMC8371368

[CR26] Mann, H. B., & Whitney, D. R. (1957). On a test of whether one of two random variables is stochastically larger than the other. *The Annals of Mathematical Statistics,**18*(1), 50–60.

[CR27] Mayrhofer, R., & Waldmann, M. R. (2015). Agents and causes: Dispositional intuitions as a guide to causal structure. *Cognitive Science,**39*(1), 65–95. 10.1111/cogs.12132 24831193 10.1111/cogs.12132

[CR28] Mestdagh, M., Verdonck, S., Meers, K., Loossens, T., & Tuerlinckx, F. (2019). Prepaid parameter estimation without likelihoods. *In PLoS Computational Biology,**15*(9), 1007181. 10.1371/journal.pcbi.1007181 10.1371/journal.pcbi.1007181 PMC6752867 31498789

[CR29] Mistry, P. K., Pothos, E. M., Vandekerckhove, J., & Trueblood, J. S. (2018). A quantum probability account of individual differences in causal reasoning. *Journal of Mathematical Psychology,**87*, 76–97. 10.1016/j.jmp.2018.09.003

[CR30] Morey, R. D., & Rouder, J. N. (2014). *BayesFactor package for R* (0.9.12–4.3). https://cran.r-project.org/web/packages/BayesFactor/index.html

[CR31] Müller, H., & Sternad, D. (2004). Decomposition of Variability in the Execution of Goal-Oriented Tasks: Three Components of Skill Improvement. *Journal of Experimental Psychology: Human Perception and Performance,**30*(1), 212–233. 10.1037/0096-1523.30.1.212 14769078 10.1037/0096-1523.30.1.212

[CR32] O’Neill, K., Henne, P., Bello, P., Pearson, J., & De Brigard, F. (2022). Confidence and gradation in causal judgment. *Cognition,**223*, 105036. 10.1016/J.COGNITION.2022.105036 35092903 10.1016/j.cognition.2022.105036

[CR33] Palminteri, S., Wyart, V., & Koechlin, E. (2017). The importance of falsification in computational cognitive modeling. *Trends in Cognitive Sciences,**21*(6), 425–433. 10.1016/j.tics.2017.03.011 28476348 10.1016/j.tics.2017.03.011

[CR34] Park, J., & Sloman, S. A. (2013). Mechanistic beliefs determine adherence to the markov property in causal reasoning. *Cognitive Psychology,**67*(4), 186–216. 10.1016/j.cogpsych.2013.09.002 24152569 10.1016/j.cogpsych.2013.09.002

[CR35] Park, J., & Sloman, S. A. (2014). Causal explanation in the face of contradiction. *Memory and Cognition,**42*(5), 806–820. 10.3758/s13421-013-0389-3 24420704 10.3758/s13421-013-0389-3

[CR36] Pearl, J. (2009). *Causality*. Cambridge University Press.

[CR37] Phillips, L. D., & Edwards, W. (1966). Conservatism in a simple probability inference task. *Journal of Experimental Psychology,**72*(3), 346–354. 10.1037/h0023653 5968681 10.1037/h0023653

[CR38] Radev, S. T., Mertens, U. K., Voss, A., Ardizzone, L., & Kothe, U. (2022). BayesFlow: Learning complex stochastic models with invertible neural networks. *IEEE Transactions on Neural Networks and Learning Systems,**33*(4), 1452–1466. 10.1109/TNNLS.2020.3042395 33338021 10.1109/TNNLS.2020.3042395

[CR39] Radev, S. T., Wieschen, E. M., & Voss, A. (2020). Amortized Bayesian inference for models of cognition. ArXiv Preprint. arXiv:2005.03899v3

[CR40] Ratcliff, R. (1978). A theory of memory retrieval. *Psychological Review,**85*(2), 59–108. 10.1037/0033-295X.85.2.59

[CR41] Ratcliff, R., Thapar, A., & McKoon, G. (2006). Aging and individual differences in rapid two-choice decisions. *Psychonomic Bulletin & Review,**13*(4), 626–635. 10.3758/BF03193973 17201362 10.3758/bf03193973 PMC2394733

[CR42] Ratcliff, R., Smith, P. L., Brown, S. D., & McKoon, G. (2016). Diffusion decision model: Current issues and history. *Trends in Cognitive Sciences,**20*(4), 260–281. 10.1016/j.tics.2016.01.007 26952739 10.1016/j.tics.2016.01.007 PMC4928591

[CR43] Rehder, B. (2014). Independence and dependence in human causal reasoning. *Cognitive Psychology,**72*, 54–107. 10.1016/j.cogpsych.2014.02.002 24681802 10.1016/j.cogpsych.2014.02.002

[CR44] Rehder, B. (2018). Beyond Markov: Accounting for independence violations in causal reasoning. *Cognitive Psychology,**103*(January), 42–84. 10.1016/j.cogpsych.2018.01.003 29522980 10.1016/j.cogpsych.2018.01.003

[CR45] Rehder, B., & Davis, Z. J. (2021). Testing a process model of causal reasoning with inhibitory causal links. *Proceedings of the Annual Meeting of the Cognitive Science Society,**43*, 43.

[CR46] Rehder, B., & Hastie, R. (2001). Causal knowledge and categories: The effects of causal beliefs on categorization, induction, and similarity. *Journal of Experimental Psychology: General,**130*(3), 323–360. 10.1037/0096-3445.130.3.323 11561914 10.1037//0096-3445.130.3.323

[CR47] Rehder, B., & Waldmann, M. R. (2017). Failures of explaining away and screening off in described versus experienced causal learning scenarios. *Memory and Cognition,**45*(2), 245–260. 10.3758/s13421-016-0662-3 27826953 10.3758/s13421-016-0662-3

[CR48] Rottman, B. M., & Hastie, R. (2014). Reasoning about causal relationships: Inferences on causal networks. *Psychological Bulletin,**140*(1), 109–139. 10.1037/a0031903 23544658 10.1037/a0031903 PMC3988659

[CR49] Rottman, B. M., & Hastie, R. (2016). Do people reason rationally about causally related events? Markov violations, weak inferences, and failures of explaining away. *Cognitive Psychology,**87*, 88–134. 10.1016/j.cogpsych.2016.05.002 27261539 10.1016/j.cogpsych.2016.05.002

[CR50] Schwarz, G. (1978). Estimating the Dimensions of a Model. *The Annals of Statistics,**6*(2), 461–464.

[CR51] Sheather, S. J., & Jones, M. C. (1991). A reliable data-based bandwidth selection method for kernel density estimation. *Journal of the Royal Statistical Society: Series B (Methodological),**53*(3), 683–690. 10.1111/J.2517-6161.1991.TB01857.X

[CR52] Sloman, S. A. (2005). *Causal models: How people think about the world and its alternatives*. Oxford University Press.

[CR53] Sloman, S. A., & Lagnado, D. (2015). Causality in thought. *Annual Review of Psychology,**66*, 223–247. 10.1146/annurev-psych-010814-015135 25061673 10.1146/annurev-psych-010814-015135

[CR54] Spirtes, P., Glymour, C., & Scheines, R. (2000). *Causation, Prediction, and Search*. MIT Press.

[CR55] Tešić, M., Liefgreen, A., & Lagnado, D. (2020). The propensity interpretation of probability and diagnostic split in explaining away. *Cognitive Psychology,**121*, 101293. 10.1016/j.cogpsych.2020.101293 32388007 10.1016/j.cogpsych.2020.101293

[CR56] Trueblood, J. S., Yearsley, J. M., & Pothos, E. M. (2017). A quantum probability framework for human probabilistic inference. *Journal of Experimental Psychology: General,**146*(9), 1307–1341. 10.1037/xge0000326 28682091 10.1037/xge0000326

[CR57] Turner, B. M., & Sederberg, P. B. (2014). A generalized, likelihood-free method for posterior estimation. *Psychonomic Bulletin and Review,**21*(2), 227–250. 10.3758/s13423-013-0530-0 24258272 10.3758/s13423-013-0530-0 PMC4143986

[CR58] van Doorn, J., Ly, A., Marsman, M., & Wagenmakers, E. J. (2020). Bayesian rank-based hypothesis testing for the rank sum test, the signed rank test, and Spearman’s ρ. *Journal of Applied Statistics,**47*(16), 2984–3006. 10.1080/02664763.2019.1709053 35707708 10.1080/02664763.2019.1709053 PMC9041780

[CR59] Van Maanen, L., Brown, S. D., Eichele, T., Wagenmakers, E.-J., Ho, T., Serences, J., & Forstmann, B. U. (2011). Neural correlates of trial-to-trial fluctuations in response caution. *Journal of Neuroscience,**31*(48), 17488–17495. 10.1523/JNEUROSCI.2924-11.2011 22131410 10.1523/JNEUROSCI.2924-11.2011 PMC6623798

[CR60] van Ravenzwaaij, D., Brown, S. D., & Wagenmakers, E.-J. (2011). An integrated perspective on the relation between response speed and intelligence. *Cognition,**119*(3), 381–393. 10.1016/j.cognition.2011.02.002 21420077 10.1016/j.cognition.2011.02.002

[CR61] van Ravenzwaaij, D., Cassey, P., & Brown, S. D. (2018). A simple introduction to Markov Chain Monte-Carlo sampling. *Psychonomic Bulletin and Review,**25*(1), 143–154. 10.3758/s13423-016-1015-8 26968853 10.3758/s13423-016-1015-8 PMC5862921

[CR62] Verdonck, S., & Tuerlinckx, F. (2013). Factoring out non-decision time in choice RT data: Theory and implications. *Psychological Review,**128*(2), 203. 10.1037/rev0000019 26641558

[CR63] Wagenmakers, E. J., & Farrell, S. (2004). AIC model selection using Akaike weights. *Psychonomic Bulletin & Review,**11*(1), 192–196. 15117008 10.3758/bf03206482

[CR64] Waldmann, M. R., Cheng, P. W., Hagmayer, Y., & Blaisdell, A. P. (2008). Causal learning in rats and humans: A minimal rational model. In N. Chater & M. Oaksford (Eds.), *The probabilistic mind. Prospects for Bayesian cognitive science* (pp. 453–484). Oxford University Press.

[CR65] Waldmann, M. R. (Ed.) (2017). *The Oxford handbook of causal reasoning*. Oxford University Press.

[CR66] Wallsten, T. S., Budescu, D. V., & Zwick, R. (1993). Comparing the calibration and coherence of numerical and verbal probability judgments. *Management Science,**39*(2), 176–190. 10.1287/mnsc.39.2.176

[CR67] Yitzhaki, S. (2003). Gini’s mean difference: A superior measure of variability for non-normal distributions. *Metron,**61*(2), 285–316. 10.2139/ssrn.301740

[CR68] Zhu, J. Q., Sanborn, A. N., & Chater, N. (2020). The Bayesian sampler: Generic Bayesian inference causes incoherence in human probability judgments. *Psychological Review,**127*(5), 719. 10.1037/rev0000190 32191073 10.1037/rev0000190 PMC7571263

