# Supplementary material for: Models of Variability in Probabilistic Causal Judgments
Source: Comput Brain Behav. 2024 Oct 8;8(1):162–88. doi: 10.1007/s42113-024-00223-7 (PMC13298624; doi:10.1007/s42113-024-00223-7)
Supplement: Supplementary file 1 — Supplementary file1 (DOCX 235 KB) [file 42113_2024_223_MOESM1_ESM.docx]

# Appendices to paper “Models of Variability in Probabilistic Causal Judgments”

## Appendix A: Treating block 1 as “burn-in”

In testing fatigue effects (see Section 2.5 in main text) we found that responses in the first block slightly differed from the blocks thereafter. This might be due to the fact that participants only settled on an interpretation of the task or on a response strategy after block 1. Here we redo figures 4 and 5, and the main analysis of the effects of Information and Direction on within-participant variability (as indexed by GMD) using only the data from the last 4 blocks. That is, we treat block 1 as “burn-in” and only look at the “stabilized” blocks.


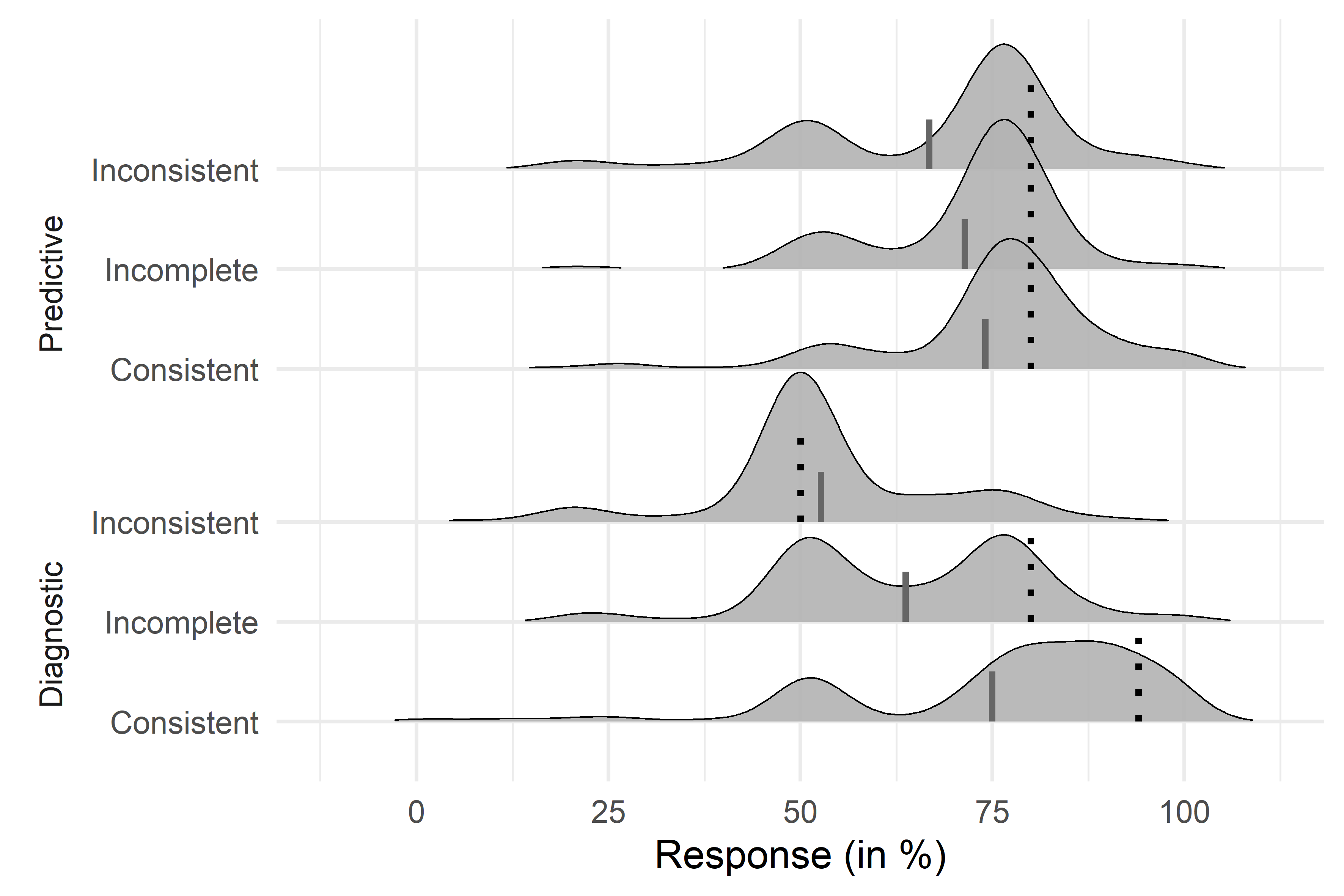


**Fig.A1** Overall response distributions per inference type excluding data from the first block. Vertical grey lines indicate mean responses. Dotted vertical black lines indicate normative response.


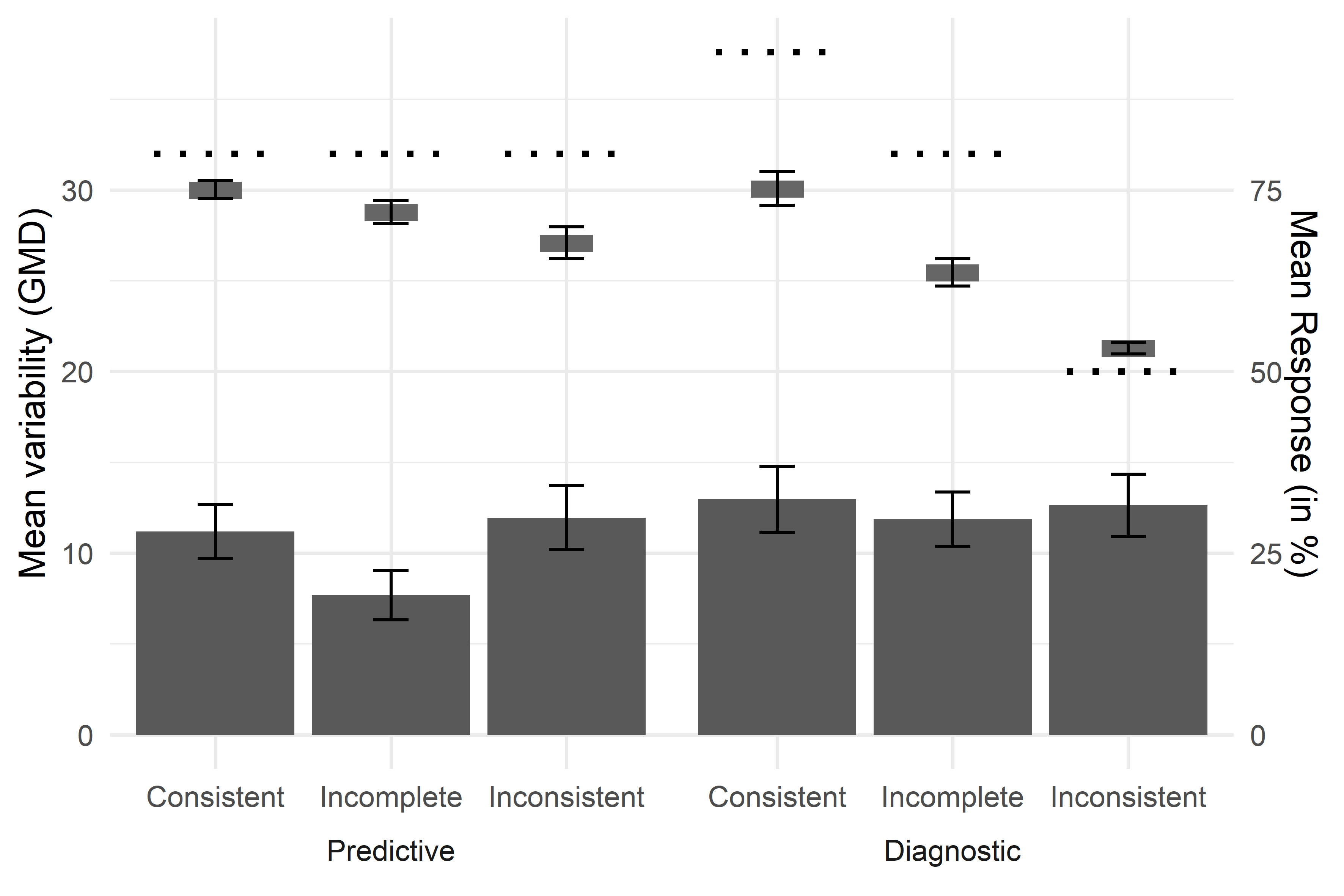


**Fig. A2** Mean and GMDs of responses per inference type excluding data from the first block. Barplot: Mean within-participant standard deviations per inference type. Floating dashes: Mean responses per inference type. Black vertical lines indicate standard error. Horizontal dotted lines indicate normative probability. GMD stands for Gini’s Mean Difference.

From Figures A1 and A2 we can see that the data excluding the first block is rather similar as when it is included (compare with Figures 4 and 5 in main text). Additionally, we redid the main analysis excluding the data from block 1. That is we retested whether variability differs over the inference types using a repeated measures ANOVA with the GMD of responses as the dependent variable and Direction (predictive vs. diagnostic) and Information (consistent, incomplete, vs. inconsistent) as factors. We find the exact same pattern of results as with the analysis reported in the main text. The main effect of Information is signiﬁcant (*F*(2,140) = 3.64, *p* = .029) reflecting that variability was lower for inferences with incomplete information (*Mean* = 9.77, *SE* = 1.4), than for those with complete information (*consistentMean* = 12.1, *SE* = 1.4, *inconsistentMean* = 12.3, *SE* = 1.4). We ﬁnd evidence of an effect of Direction (*F*(1,140) = 6.84, *p* = .0099): Variability was higher for diagnostic inferences (*Mean* = 12.5, *SE* = 1.4) than for predictive inferences (*Mean* = 10.3, *SE* = 1.4). There was no evidence for a Direction × Information interaction (*F*(2,140) = 1.49, *p* = .228). We conclude that while it might indeed be true that participants settled on an interpretation or response strategy after block 1, this does not seem to affect our results.

## Appendix B: remaining sources of dependence

Here we look into whether possible remaining sources of dependence could influence our results. Specifically, we look into the effect of asking participants to judge the absence versus presence of an effect, and into the effect of switching around the state of the two symmetrical causal effects (X_1_ and X_2_, see experimental design in Section 2.6 of the main text).

Figure B1 shows the response distributions for each inference type separated by whether the X_1_ or X_2_ was queried (diagnostic inferences) or whether the X_1_ or X_2_ was present in the conditional statement (predictive inferences). There seem to be no systematic effects due to changing around X_1_ and X_2_. To test the effect of changing around X_1_ and X_2_ we used a repeated measures ANOVA with the responses as dependent variable, and Direction, Information, and X_1_ versus X_2_ as factors including all their interactions. We find no significant interaction with the variable indicating X_1_ vs X_2_ (Information: *F*(2,253) = 0.096, *p* = .909, *BF_10_* = 0.0820; Direction: *F*(1,253) = 0.097, *p* = .755, *BF_10_* = 0.192), nor a main effect (*F*(1,254) = 0.723, *p* = .40, *BF_10_* = 0.200).


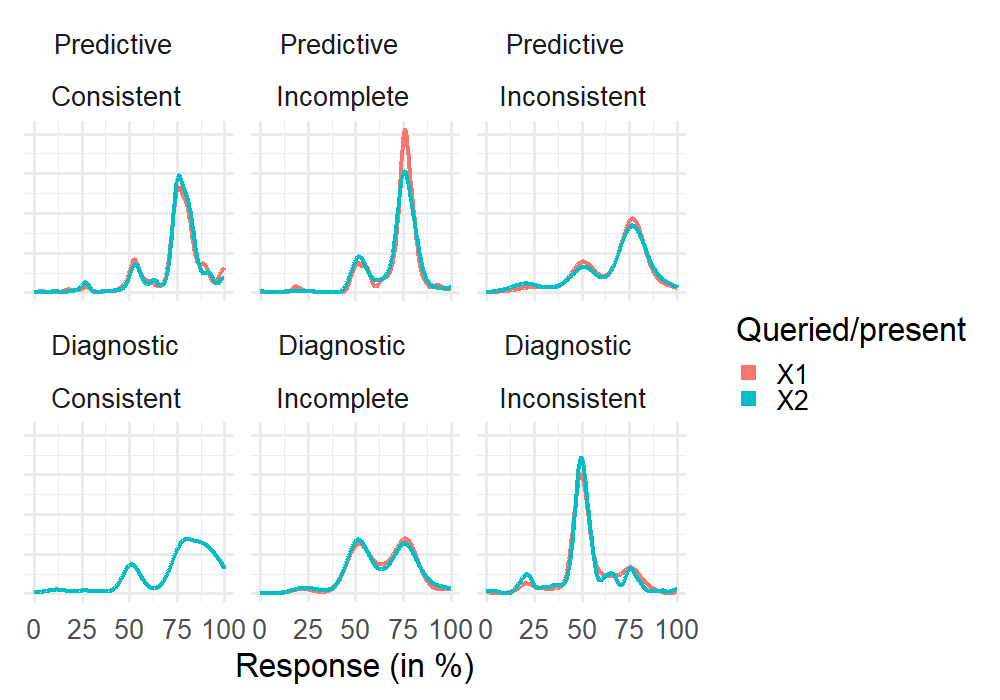


**Fig. B1** Response distributions per inference type for all participants. Separate colored lines indicate whether the X_1_ or X_2_ was queried (diagnostic inferences) or whether the X_1_ or X_2_ was present in the conditional statement (predictive inferences).

Figure B2 shows the response distributions for each inference type separated by whether the presence or absence of a variable was queried. The main worry concerning this is that participants might not have understood that the absence was queried and so would respond on the opposite side of the response scale. Figure C1 shows that this is not the case, almost all responses are above 50%. There are some differences between the distributions, specifically for the predictive consistent and the diagnostic inconsistent inferences, but overall the distributions are rather similar. To test the effect of querying presence versus absence we used a repeated measures ANOVA with the responses as dependent variable, and Direction, Information, and whether presence was queried included as factors including all their interactions. We find a significant interaction effect of whether presence was queried with the Information factor (*F*(2,311) = 12.8, *p* < .001, *BF_10_* = 586), but no main effect of whether presence was queried (*F*(1,311) = 0.91, *p* = .34, *BF_10_* = 0.169). Does this impact our results? If we add the factor whether presence was queried to our ANOVA on within-participant variability (as indexed by GMD) the effects of information (*F*(2,313) = 7.86, *p* < .001, *BF_10_* = 49) and reasoning direction (*F*(1,313) = 6.35, *p* = .012, *BF_10_* = 2.05) remain the same, indicating that our findings in Section 2 with regard to within-participant variability of the main text are not due to whether presence or absence is queried.


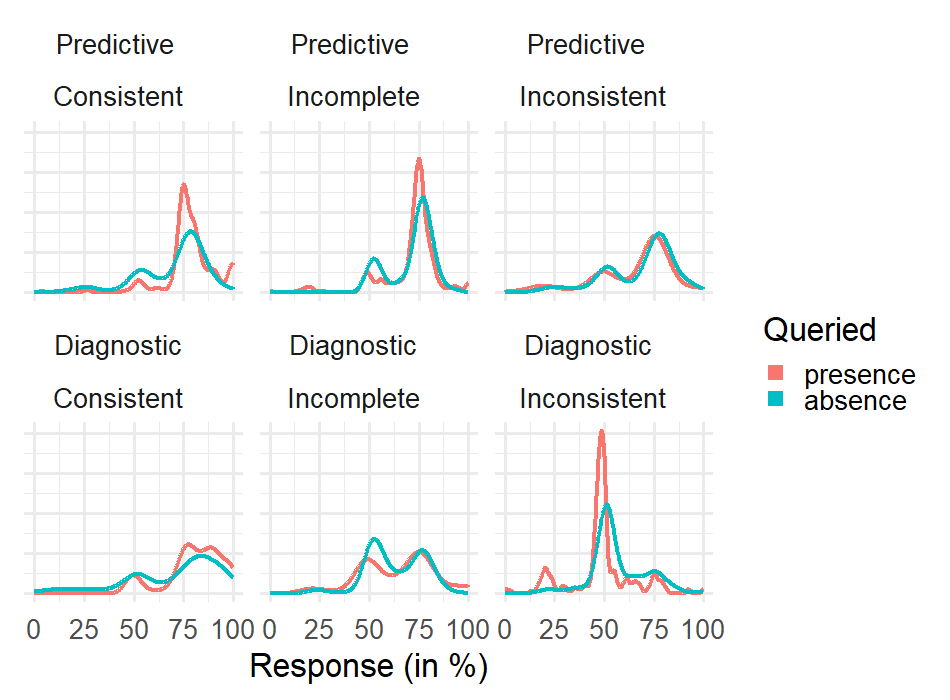


**Fig. B2**. Response distributions per inference type for all participants. Separate colored lines indicate whether the query referred to the presence or absence of a causal variable. Responses to queries of the absence of a variable are flipped across the response scale using 1 – judgment (see main text).
